# Invasive validation of BSE-2024 vs. BSE-2013 using LVEDP and LV pre-A pressure in patients undergoing cardiac catheterization: a multicenter study with a complementary algorithm

**DOI:** 10.1186/s44156-026-00132-4

**Published:** 2026-07-27

**Authors:** Amer Barakat, Ali Khaddam, Mhd Ameen Alkhatib, Rami Alsaadi, Ahmad Rasheed Alsaadi

**Affiliations:** Department of Cardiology, Damascus University, Fayez Mansour Street, Damascus, Syria

**Keywords:** Diastolic function, LV filling pressure, BSE, Proposed algorithm, LA strain

## Abstract

**Objective:**

To invasively validate the 2024 and 2013 British Society of Echocardiography (BSE) recommendations for diastolic function (DF) and left ventricular filling pressure (LVFP) detection using LV end-diastolic pressure (LVEDP) ≥ 16 mmHg and LV pre-A > 15 mmHg in patients undergoing left heart catheterization (LHC) and to propose a complementary algorithm to improve diagnostic performance.

**Methods:**

In this prospective multicenter study, 716 patients in sinus rhythm underwent echocardiography within 120 min before clinically indicated LHC (401 derivations and 315 temporally independent validation cohort). Patients with conditions that could interfere with the reliability of the algorithms were excluded. Both guidelines and proposed algorithms estimated DF and LVFP. The diagnostic classifications were compared with invasively-measured LVEDP and LV pre-A pressure.

**Results:**

BSE-2024 classified patients as (0.7%) indeterminate, (49.1%) normal and (50.1%) impaired DF, compared with (60.6%), (14.2%) and (25.2%), respectively, using BSE-2013. After excluding indeterminate cases, area under the curve (AUC) for detecting LVFP was comparable (e.g. for LVEDP, BSE-2024: 0.602 vs. BSE-2013: 0.630, *P* = 0.459). Updated guidelines achieved higher specificity for LVEDP (93.5% vs. 81.9%) but substantially lower sensitivity (27.8% vs. 44.2%), similar to that for LV pre-A (specificity: 92.1% vs. 79.4% and sensitivity 38.2% vs. 50.8%). BSE-2024 reclassified (82.3%) of indeterminate cases and (60.8%) of elevated LVFP under BSE-2013 as normal, with no significant change in overall reclassification (NRI: -0.056, *P* = 0.41; IDI: -0.056, *P* = 0.40). The proposed algorithm performance results were {LVEDP: (78.8%) sensitivity and (79.8%) specificity, while LV pre-A: (85.5%) sensitivity and (67.3%) specificity}. The independent validation cohort confirmed the robustness and reproducibility of the complementary algorithm {LVEDP: (88.3%) sensitivity and (87.9%) specificity and LV pre-A: (95.7%) sensitivity and (64.3%) specificity}.

**Conclusions:**

BSE-2024 markedly reduced indeterminate classifications but showed reduced sensitivity and modest diagnostic discrimination against invasive reference standards. Comparisons should be interpreted cautiously because BSE-2013 excludes many indeterminate cases from performance analyses, whereas BSE-2024 retains nearly all patients. The complementary algorithm provided a more balanced diagnostic profile and warrants further external validation.

**Supplementary Information:**

The online version contains supplementary material available at 10.1186/s44156-026-00132-4.

## Introduction

Accurate assessment of Left ventricular (LV) diastolic function (DF) and elevated left ventricular filling pressure (LVFP) is clinically important but remains challenging in clinical practice [1]. Echocardiography [2, 3] is the most widely used non-invasive technique [4–7] for detecting LVFP, using hemodynamic assessment as the reference standard.

To address this challenge, the British Society of Echocardiography (BSE) first published guidelines in 2013 [6]. Although practical, these were criticized for classifying a substantial proportion of patients as indeterminate.

In 2024, the BSE introduced updated recommendations [8] to improve feasibility and diagnostic clarity, incorporating global longitudinal strain (GLS), left atrial reservoir strain (LASr) [9–11] and age-specific tissue Doppler e′ cut-offs alongside conventional indices [12]. BSE-2024 introduced separate algorithms for heart failure patients in sinus rhythm with preserved (HFpEF), reduced (HFrEF) ejection fraction, including a dedicated algorithm for patients with atrial fibrillation (AF) [8].

LVFP is a broad physiological concept that encompasses several invasive pressure measurements. For example, mean pulmonary capillary wedge pressure (PCWP), mean left atrial pressure (LAP), left ventricular end-diastolic pressure (LVEDP) and LV pre-A pressure, each reflecting a different aspect of cardiac filling physiology when used as reference standards in validation studies [1].

LVEDP is the most frequently used reference and represents ventricular pressure at the end of diastole and reflects LV preload [8, 13].

LV pre-A pressure is an increasingly accepted invasive reference standard [1, 13–15]. It is measured immediately before atrial contraction and is therefore less affected by active atrial filling, making it a closer surrogate for mean LAP and PCWP.

**Fig. 1:**
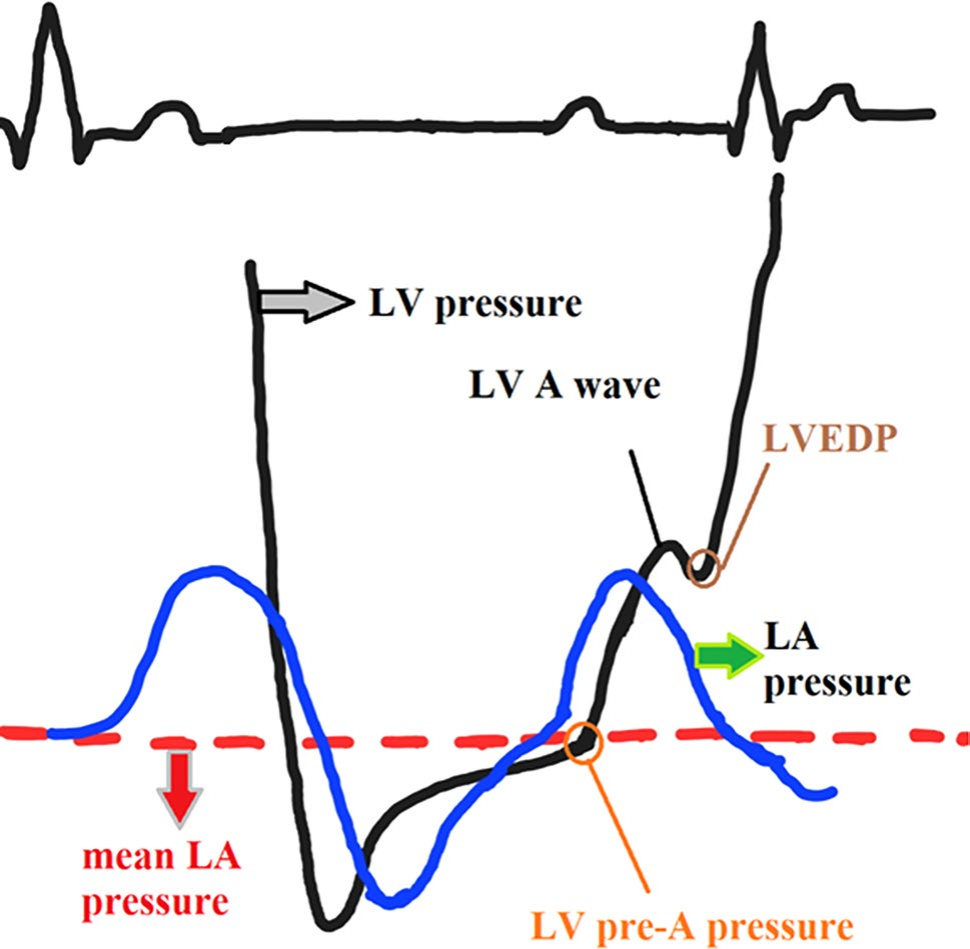
Illustrative figure for invasive left ventricular filling pressure measurements. Blue curve: Left atrial (LA) pressure (LAP) tracing, middle black curve: LV pressure tracing and position of LVEDP and LV pre-A pressure, red line: mean arterial pressure. Putting these curves together shows correlation of LV pre-A pressure and mean LAP

Overall, LAP and LVFP are closely related but not synonymous (Fig. 1). Nevertheless, contemporary echocardiographic recommendations commonly use LAP as a non-invasive surrogate for LVFP. Therefore, the term LAP is used to refer to the non-invasive estimation of LVFP according to echo-guideline algorithms.

Despite these advances, invasive validation of BSE-2024 recommendations remains limited. Additionally, a direct comparison with BSE-2013 would be beneficial. This study addresses that gap by providing a prospective multicenter invasive comparison of both recommendations, proposing a complementary LASr/PV–based algorithm to further improve LVFP detection.

## Methods

### Study design and population

This prospective multicenter study was conducted across three university hospitals comprising five cardiac catheterization laboratories. Consecutive eligible patients referred for clinically indicated cardiac catheterization, with or without subsequent coronary intervention, were screened between June 2024 and June 2025. All participants underwent comprehensive transthoracic echocardiography (TTE) within 120 min before invasive assessment. Of 809 patients screened, 716 fulfilled the eligibility criteria and were included in the study. The study population comprised a derivation cohort (*n* = 401) and a temporally independent validation cohort (*n* = 315).

#### Derivation and validation cohort

The derivation cohort included patients recruited during the initial phase of the study, whereas the validation cohort consisted of patients prospectively enrolled during a subsequent period, ensuring complete temporal separation without patient overlap.

Both cohorts were recruited from the same participating centers, met identical inclusion and exclusion criteria and underwent identical echocardiographic and invasive acquisition protocols.

The complementary algorithm was developed using the derivation cohort and subsequently applied without modification to the independent validation cohort.

Exclusion criteria included refusal to participate, atrial fibrillation or other significant arrhythmias, cardiac arrest, constrictive pericarditis, moderate-to-severe valvular heart disease (including severe mitral annular calcification), prosthetic heart valves, paced wide-QRS rhythms affecting Doppler timing, congenital heart disease, poor acoustic windows, inability to undergo catheterization or invasive LVFP assessment and hemodynamic instability. These criteria were consistent with the corresponding guidelines and previous invasive validation studies [8, 13–15].

#### Catheterization indication and invasive pressure measurements conditions

Patients underwent left heart catheterization for established clinical indications in accordance with contemporary guidelines, including the evaluation of acute or chronic coronary syndromes, unexplained dyspnea after inconclusive non-invasive investigations, pre-operative cardiac assessment and evaluation of heart failure or suspected structural or hemodynamic abnormalities [16–18]. All catheterization procedures were performed as part of routine clinical care rather than solely for research purposes, providing a real-world cohort undergoing invasive cardiovascular assessment [16, 17].

Invasive LVFP measurements are not routinely obtained during every catheterization procedure [1, 19]. But when feasible, safe and under standardized conditions, guidelines state these measurements provide robust physiological information [1, 16, 19].

In the present study, invasive pressure measurements were obtained only when considered clinically feasible, provided that no contraindications or additional procedural risks were present and after written informed consent had been obtained.

### Echocardiography

All participating centers used GE Vivid T9 ultrasound systems (GE Healthcare, Horten, Norway) and strain analyses were performed using EchoPAC software (GE Healthcare). Image acquisition and analysis followed standardized protocols based on current recommendations to ensure consistency across centers [8, 13].

Blinded to the invasive hemodynamic findings, TTE was performed by certified cardiologists with at least five years of experience in advanced echocardiography.

Standard echocardiographic measurements included transmitral E/A ratio, E-wave deceleration time (EDT), septal and lateral tissue Doppler e′ velocities with averaged E/e′. Left atrial volume index (LAVi), tricuspid regurgitation (TR) velocity, pulmonary artery systolic pressure (PASP), PV: systolic-to-diastolic (S/D) flow ratio and the difference between atrial reversal duration and mitral A-wave duration (Ar–A) were also obtained.

Left ventricular global longitudinal strain (GLS) was measured from standard apical four-, two- and three-chamber views using 2D speckle-tracking echocardiography. Image quality was optimized to ensure accurate endocardial tracking and GLS was calculated as the average peak systolic strain across all LV segments [8, 13, 20].

Left atrial reservoir strain (LASr) was assessed during the atrial reservoir phase from apical four- and two-chamber views. The left atrial endocardial border was manually traced while excluding PV ostia and measurements were averaged over multiple cardiac cycles to improve reproducibility.

#### Echocardiography reliability and inter-center variability

To assess reproducibility, a random sample of 50 studies was independently reanalyzed by a second observer blinded to both the initial measurements and invasive findings. Inter-observer agreement was excellent, with intraclass correlation coefficients ranging from 0.84 to 0.91 for the principal echocardiographic parameters, including GLS (0.89) and LASr (0.91).

Because all participating centers used the same ultrasound platform and analysis software, inter-vendor variability was minimized.

### Invasive reference standard

The participating centers used Allura (Philips Healthcare, The Netherlands), Azurion (Philips Healthcare, The Netherlands), and Infinix Celeve (Canon Medical Systems, Japan) angiography platforms.

All sites followed a standardized protocol for invasive pressure acquisition. Interventional cardiologists received protocol training before study initiation and were blinded to the echocardiographic findings. Meanwhile, pressure tracings were periodically reviewed in a blinded fashion.

LVFP was measured during cardiac catheterization using a 6-Fr pigtail catheter introduced through either femoral or radial arterial access. Pressure tracings were acquired with a fluid-filled transducer zeroed at the mid-axillary line before contrast administration. All measurements were obtained at end-expiration, averaged over at least three consecutive cardiac cycles and recorded using a sweep speed of 100 mm/s with a maximum pressure scale of 40 mmHg.

LVEDP was measured at the nadir of the LVFP tracing immediately following atrial contraction. LV pre-A pressure was defined as the ventricular pressure immediately before the onset of the atrial contraction wave on the pressure tracing. An illustrative example of both measurements is provided in Fig. 1, consistent with the American Society of Echocardiography and European Association of Cardiovascular Imaging (ASE/EACVI) recommendations [1].

Elevated LVEDP was defined as ≥ 16 mmHg, whereas elevated LV pre-A pressure was defined as > 15 mmHg [8, 13]. An exploratory composite reference standard was also evaluated, defining elevated LVFP as the presence of either LVEDP ≥ 16 mmHg or LV pre-A pressure > 15 mmHg.

#### Invasive measurements reliability and inter-center variability

Reproducibility analysis demonstrated excellent agreement for both LVEDP and LV pre-A measurements, with intraclass correlation coefficients of 0.90 and 0.91, respectively. Bland–Altman analysis showed minimal inter-center bias (mean difference, 0.8 mmHg; 95% limits of agreement, − 3.2 to 4.8 mmHg).

To further account for potential center-related variability, mixed-effects regression models were incorporated into the primary analysis. Sensitivity analyses excluding individual centers produced comparable results, confirming the robustness of the study findings.

### Classification by BSE-2013 and BSE-2024

Each examination was independently and strictly classified according to both original BSE recommendations [6, 8]. According to the BSE-2013, DF was categorized as normal, impaired (Grades I–III) or indeterminate and LVFP was classified as normal, elevated, or indeterminate.

Similar classifications were followed applying the updated recommendations but it should be noted that diastolic dysfunction grading no longer exists in BSE-2024.

### Complementary proposed algorithm

**Fig. 2:**
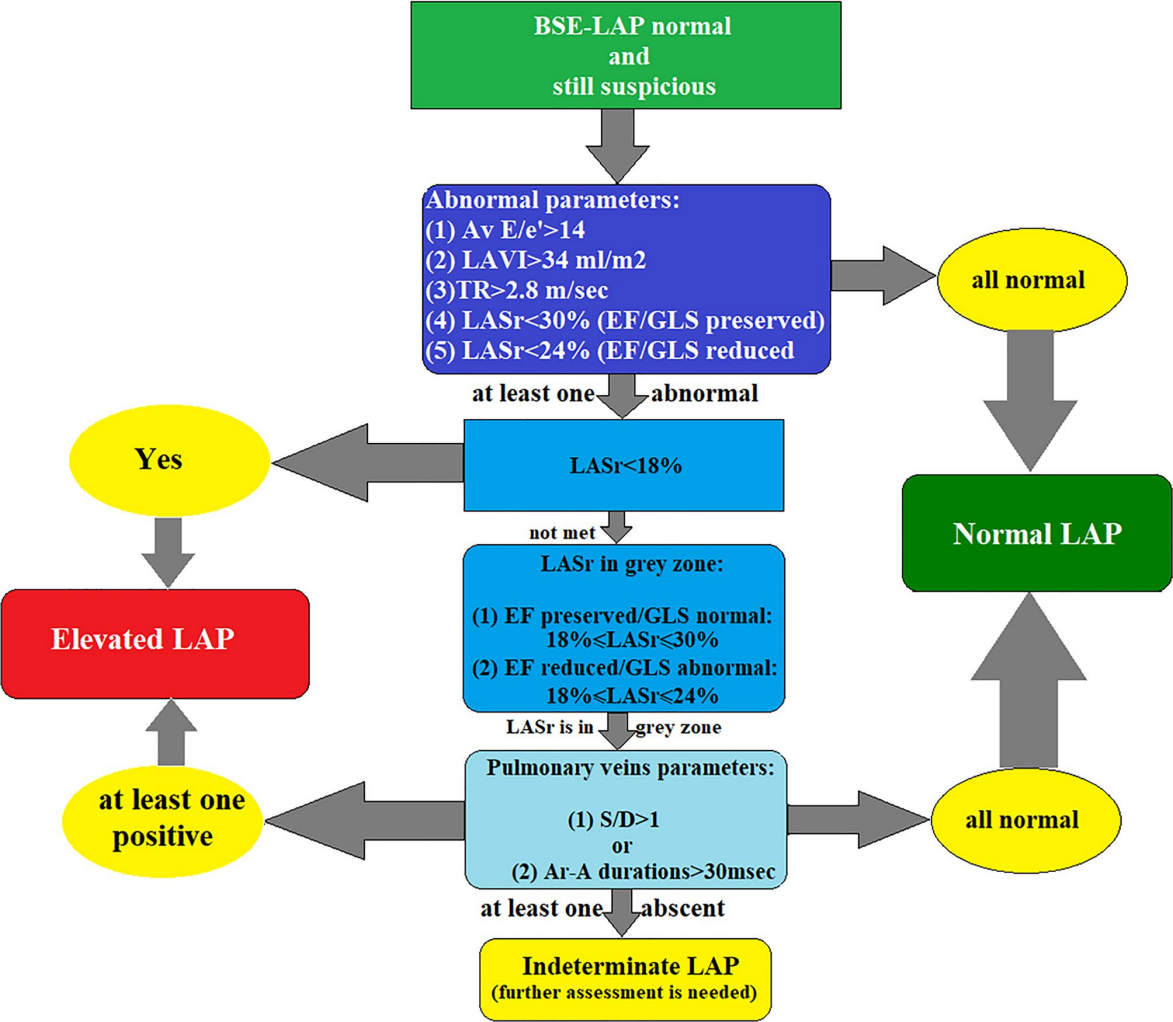
A complementary proposed algorithm to BSE-2024

To explore whether diagnostic accuracy could be further improved, this study developed an exploratory, complementary two-step escalation layer that was triggered only if BSE-2024 classified LAP as normal, while elevated and indeterminate classifications were left unchanged (Fig. 2):

Step 1 – Abnormal cut-offs (any abnormal or any missing ⇒ Step 2):

(1) TR > 2.8 m/s; (2) avg E/e′ >14; (3) LAVi > 34 mL/m²; (4) LASr < 30% if LVEF ≥ 50%; (5) LASr < 24% if LVEF < 50%.

If all five normal and none missing → retain normal LAP.

Step 2 – LASr-anchored adjudication + PV tie-breakers:

- If LASr > 30% (LVEF ≥ 50%) or > 24% (LVEF < 50%) → Normal LAP.
- If LASr < 18% (any LVEF) → Elevated LAP.
- If LASr in the grey-zone (18–30% or 18–24%), then use PV: S/D > 1 and Ar–A > 30 ms to confirm normal LVFP; any abnormal PV index reclassifies to elevated LVFP; if PV data is not completely available without any abnormal indices → Indeterminate.

These thresholds were chosen based on recent guidelines and invasive studies [1, 6, 8, 9, 13].

### Outcomes and statistical analysis

The primary outcome was the diagnostic performance of each algorithm in detecting elevated LVEDP, elevated LV pre-A pressure and the exploratory composite LVFP endpoint. After excluding indeterminate cases, diagnostic performance was assessed using sensitivity, specificity, positive predictive value (PPV), negative predictive value (NPV), diagnostic accuracy and the area under the receiver operating characteristic curve (AUC).

Secondary outcomes included agreement (Cohen’s κ), reclassification using the net reclassification improvement (NRI) and integrated discrimination improvement (IDI) for incremental value and model calibration assessed by calibration intercept, calibration slope and the Brier score.

Continuous variables were compared using Student’s t-test or the Mann–Whitney U test for non-normal distributions, as appropriate, whereas categorical variables were compared using the χ² or Fisher’s exact test. ROC curves were compared using DeLong’s test. All statistical tests were two-sided, and a P value < 0.05 was considered statistically significant. Statistical analyses were performed using SPSS v27 (IBM Corp., Armonk, NY, USA) and R v4.2.

## Results

### Baseline characteristics

#### Derivation cohort

Patients with elevated LV pre-A pressure had significantly lower LV systolic function, reflected by reduced LVEF and less negative longitudinal strain (GLS = − 15.37 ± 1.42% vs − 17.57 ± 1.76%; P < 0.0001). They also significantly exhibited larger LAVi, higher average E/e’, longer (AR-A) duration and lower LA strain values (LASr = 24.45 ± 8.99% vs. 30.05 ± 10.73%; *P* < 0.0001) (Table 1).

**Table 1:** Baseline characteristics by LV pre-A > 15 mmHg

Descriptive Variables			
Variable	LV pre-A ≤ 15 (*n* = 269)	LV pre-A > 15 (*n* = 132)	P-value
HTN	151 (56.1%)	64 (48.5%)	0.1813
DM	122 (45.4%)	98 (74.2%)	< 0.0001
IHD	116 (43.1%)	81 (61.4%)	0.0009
Female	121 (45.0%)	63 (47.7%)	0.6804
**Continuous Variables**			
Variable	LV pre-A ≤ 15 Mean ± SD	LV pre-A > 15 Mean ± SD	P-value
Age (years)	55 ± 10	56 ± 7	0.3728
EF (%)	58 ± 9	53 ± 9	< 0.0001
LAVI (mL/m²)	20.98 ± 7.8	24.52 ± 9.28	0.0002
Av E/e′	7.86 ± 3.41	8.92 ± 4.31	0.0141
LA Strain (%)	30.05 ± 10.73	24.45 ± 8.99	< 0.0001
GLS (%)	-17.57 ± 1.76	-15.37 ± 1.42	< 0.0001
E/A (ratio)	1.03 ± 0.33	1.14 ± 0.42	0.0217
DT (ms)	206.4 ± 50.54	189.07 ± 48.2	0.0010
TR Velocity (m/s)	1.54 ± 0.72	1.99 ± 0.94	0.0005
PASP (mmHg)	23.48 ± 6.12	37.41 ± 17.22	0.0006
S/D Ratio	1.48 ± 0.38	1.26 ± 0.38	< 0.0001
Ar-A duration (ms)	13.07 ± 22.28	27.26 ± 16.61	< 0.0001
BMI (kg/m²)	27.98 ± 4.91	28.63 ± 4.77	0.1920
BSA (m²)	1.92 ± 0.19	1.93 ± 0.19	0.7390

LVEDP: Left Ventricular End-Diastolic Pressure, SD: standard deviation, EF: Ejection Fraction, LAVI: Left Atrial Volume Index, average E/e′: Mitral inflow E to Tissue Doppler e′ velocity (averaged), LA strain: Left Atrial Reservoir Strain, GLS: Global Longitudinal Strain, E/A: Ratio of early to late mitral inflow velocity, DT: Deceleration Time of E mitral flow, TR: Tricuspid Regurgitation velocity, PASP: Pulmonary Artery Systolic Pressure, S/D: Pulmonary Vein Systolic/Diastolic Ratio, Ar-A: Difference in duration between pulmonary vein Ar and mitral A wave, BMI: Body Mass Index and BSA: Body Surface Area, HTN: Hypertension, DM: diabetes mellitus and IHD: ischemic heart disease

Elevated LVEDP showed consistent patterns (Supplementary Table 1). Hemodynamic variables remained comparable between echocardiography and catheterization (Supplementary Table 2), while comorbidities and medication use are summarized in (Supplementary Table 3).

#### Validation cohort

This cohort included 315 patients (41.3% women; mean age 56 ± 9 years), with a mean BSA of 1.90 ± 0.15 m², BMI of 27.85 ± 4.86 kg/m², EF of 56 ± 10%, diabetes in 61.27% and hypertension in 62.86%.

Likewise, elevated LVEDP was associated with significantly lower LASr, lower S/D ratio (0.81 ± 0.32 vs. 1.49 ± 0.31) and less negative longitudinal strain (GLS = -15.81 ± 2.11% vs. -17.69 ± 1.55%), together with higher PASP and TR velocity and longer Ar–A duration (29.59 ± 23.78 vs. 4.90 ± 26.58 milliseconds) (all *p* < 0.001). Average E/e′ also increased significantly, although the absolute difference was modest in p value (9.91 ± 3.35 vs. 9.32 ± 3.35: *p* = 0.027). Consistent results were observed when patients were stratified by LVpre-A.

### Feasibility of PV and TR parameters

PV Doppler measurements (Supplementary Fig. 1) demonstrated good feasibility. In the derivation cohort, S/D ratio was successfully obtained in 83.3% of patients and (AR–A) duration in 80.6%. At least one PV parameter was available and clinically informative in 86.7% of patients, allowing the complementary algorithm to reach a definitive classification. The remaining 13.3% were classified as indeterminate because PV data were unavailable.

The validation cohort maintained comparable feasibility. S/D ratio and (AR–A) duration were successfully acquired in 86.7% and 83.2% of patients, respectively. Overall interpretability reached 90.8%, while the remaining 9.2% of examinations were conservatively classified as indeterminate.

TR velocity (Supplementary Fig. 2) was measurable in 79.3% of patients in the derivation and 80.6% in the validation cohort. When TR velocity was unavailable, the corresponding criterion in Step 1 was considered not fulfilled and patients proceeded to Step 2, where classification relied on the remaining available echocardiographic parameters in accordance with the predefined algorithm [13].

### Diastolic function classification BSE-2024 vs. BSE-2013

BSE-2024 markedly reduced the proportion of indeterminate DF classifications, from 60.6% with BSE-2013 to 0.7% (Supplementary Fig. 3). Consequently, patients were redistributed into more balanced normal DF and impaired categories (49.1% and 50.1%, respectively), compared with 14.2% and 25.2% using BSE-2013 (Table 2).

**Table 2:** Comparison of diastolic function classification between BSE-2024 and BSE-2013

Guideline	Indeterminate	Normal	Impaired	
BSE-2024	3 (0.7%)	202 (49.1%)	196 (50.1%)	
BSE-2013	243 (60.6%)	57 (14.2%)	101 (25.2%)	Grade I: 12.5% Grade II: 12.0% Grade III: 0.7%
P-value	< 0.0001	< 0.0001	< 0.0001	

BSE = British Society of Echocardiography

Similar patterns emerged for LVFP classification (Supplementary Fig. 4). BSE-2024 classified substantially more patients as having normal (81.5%) or elevated (17.7%) LVFP than BSE-2013 (26.7% and 12.7%, respectively), primarily by minimizing the indeterminate category (Table 3).

**Table 3:** Comparison of LAP category distribution between BSE-2013 and BSE-2024 guideline

Guidelines	Normal LAP	Elevated LAP	Indeterminate LAP
BSE-2024	327 (81.5%)	71 (17.7%)	3 (0.7%)
BSE-2013	107 (26.7%)	51 (12.7%)	243 (60.6%)
p-value	< 0.0001	*P* = 0.06	< 0.0001

BSE = British Society of Echocardiography, LAP: left atrial pressure

### Invasive validation (BSE-2024 vs. BSE-2013)

**Fig. 3:**
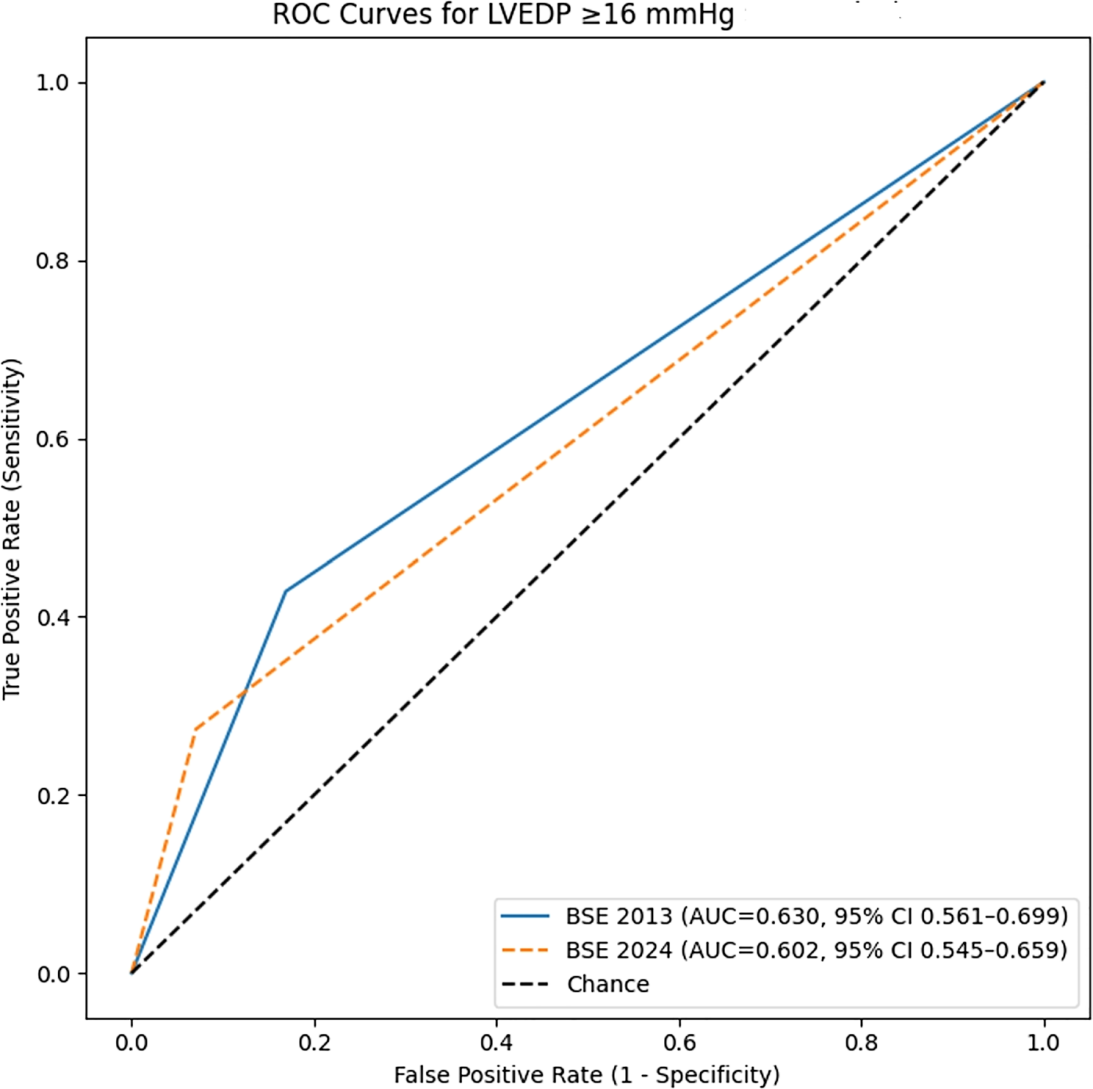
Area under the curve (AUC) comparison between BSE-2013 and BSE-2024 for LVEDP≥16mmHg. *: BSE-2013 data are derived after excluding approximately 60% of (indeterminate cases), whereas the BSE-2024 algorithm retained nearly all patients. therefore, direct comparisons of performance metrics should be interpreted with caution

**Fig. 4:**
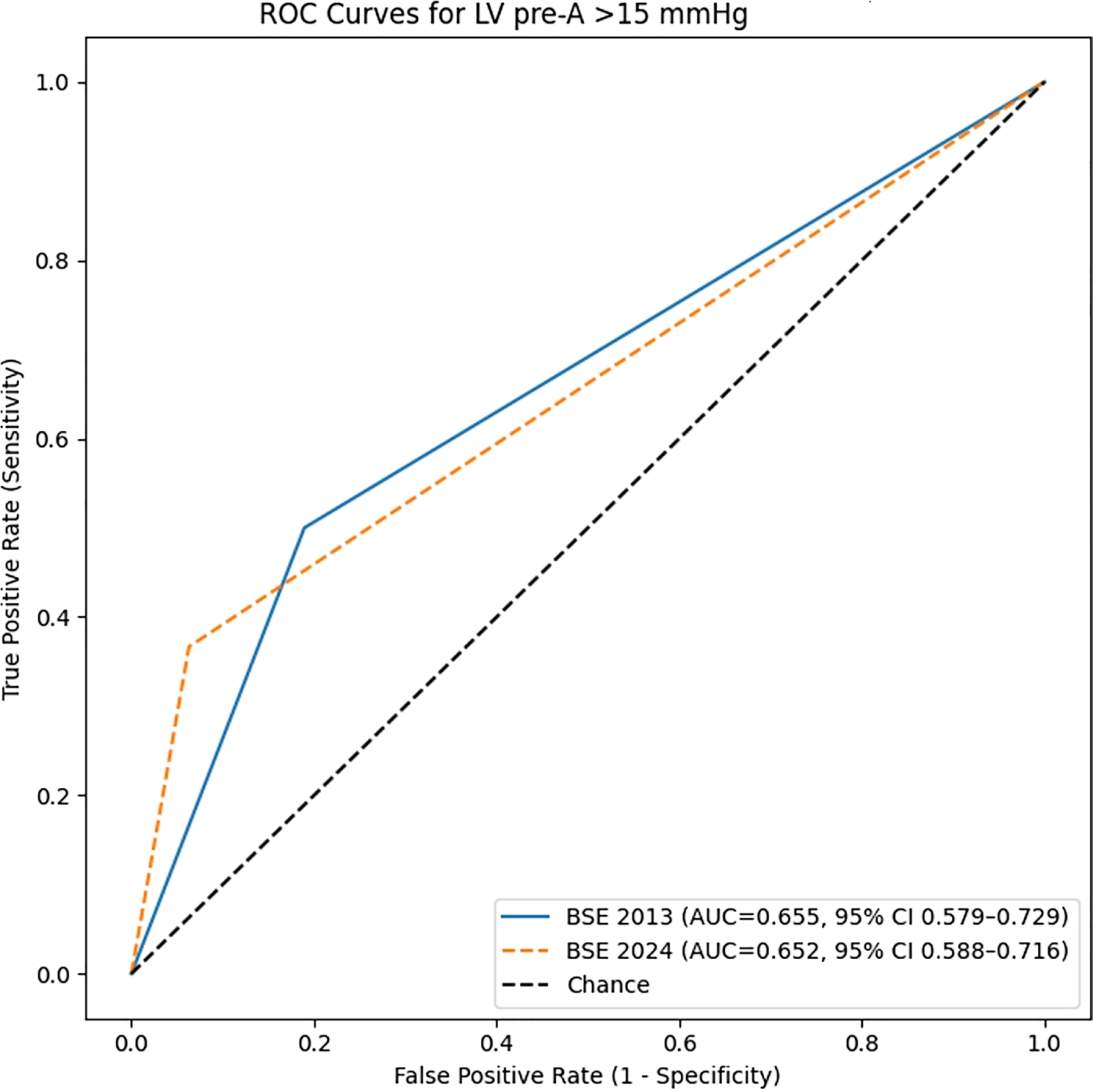
Area under the curve (AUC) comparison between BSE-2013 and BSE-2024 for LV pre-A>15mmHg. *: BSE-2013 data are derived after excluding approximately 60% of (indeterminate cases), whereas the BSE-2024 algorithm retained nearly all patients. therefore, direct comparisons of performance metrics should be interpreted with caution

After exclusion of indeterminate cases, given the substantial difference in the proportion of indeterminate cases between BSE-2013 and BSE-2024, both recommendations demonstrated modest discrimination for detecting elevated invasive LVFP (Table 4). Regarding elevated LVEDP, AUC was 0.602 for BSE-2024 and 0.630 for BSE-2013 (*P* = 0.459). Likewise, differences in AUCs for elevated LV pre-A pressure were also not significant (Figs. 3 and 4; *P* = 0.921).

**Table 4:** Diagnostic performance and AUC comparison between BSE-2013 and BSE-2024 for predicting invasive LVFP*

Pressure wave	Guidelines	AUC* (95% CI)	Sensitivity	Specificity	PPV	NPV	Accuracy	*P*-value AUCs*
LV pre-A > 15 mmHg	BSE-2024	0.652 (0.588–0.716)	38.2%	92.1%	70.4%	75.2%	74.4%	0.9208
	BSE-2013	0.655 (0.579–0.729)	50.8%	79.4%	60.8%	72%	68.4%	
LVEDP ≥ 16 mmHg	BSE-2024	0.602 (0.545–0.659)	27.8%	93.5%	83.1%	53.2%	58.5%	0.4592
	BSE-2013	0.630 (0.561–0.699)	44.2%	81.9%	74.5%	55.1%	61.4%	
LVFP either positive	BSE-2024	0.622 (0.569–0.675)	29.5%	96.1%	90.1%	53.2%	59.8%	0.779
	BSE-2013	0.632 (0.562- 0.7)	43.8%	82.6%	76.5%	53.3%	60.8%	

BSE = British Society of Echocardiography, LVEDP: left ventricular end diastolic pressure, LVFP: left ventricular filling pressure, AUC: Area under the curve, NPV: negative predictive value and PPV: positive predictive value. *: BSE-2013 data are derived after excluding approximately 60% of (indeterminate cases), whereas the BSE-2024 algorithm retained nearly all patients. therefore, direct comparisons of performance metrics should be interpreted with caution

Furthermore, BSE-2024 algorithm consistently achieved higher specificity but lower sensitivity across the evaluated invasive reference standards, whereas overall diagnostic accuracy remained consistent (Supplementary Figs. 5–7).

Notably, 82.3% of patients classified as indeterminate by BSE-2013 were reclassified as normal LVFP, while the other 17.7% were considered elevated with BSE-2024. Similarly, 60.8% of BSE-2013 elevated LVFP were redistributed as normal under BSE-2024. However, the majority (89.7%) remained classified as having normal LVFP by both guidelines (Table 5). Agreement between the two recommendations remained low (Supplementary Table 4).

**Table 5:** Reclassification BSE-2013 → BSE-2024

BSE-2013 LAP	2024 Normal LAP	2024 Elevated LAP	2024 Indeterminate
Normal (*n* = 107)	96	11	0
Elevated (*n* = 51)	31	17	3
Indeterminate (*n* = 243)	200	43	0

BSE = British Society of Echocardiography, LAP: left atrial pressure

Overall reclassification was not significantly improved (overall NRI − 0.056, *P* = 0.41; IDI − 0.056, *P* = 0.40), indicating that BSE-2024 mainly altered classification without significantly changing overall discrimination against invasive reference standards (Table 6).

**Table 6:** NRI and IDI (BSE-2024 vs. BSE-2013) for different invasive pressure wave

pressure wave	Metric	Estimate	95% CI	*p*-value
LVEDP ≥ 16 mmHg	Event NRI	−0.155	−0.280 to − 0.032	-
	Non-event NRI	+ 0.099	+ 0.014 to + 0.190	-
	Total NRI	−0.056	−0.210 to + 0.099	0.41
	IDI (Δ discrimination slope)	−0.056	−0.210 to + 0.099	0.40
LV pre-A>15mmHg	Event NRI	−0.133	−0.298 to + 0.033	-
	Non-event NRI	+ 0.126	+ 0.051 to + 0.208	-
	Total NRI	−0.007	−0.189 to + 0.172	0.94
	IDI (Δ discrimination slope)	−0.007	−0.189 to + 0.172	0.93

NRI Net Reclassification Index and IDI: Integrated Discrimination Improvement

### Complementary algorithm (derivation and validation cohort)

**Fig. 5:**
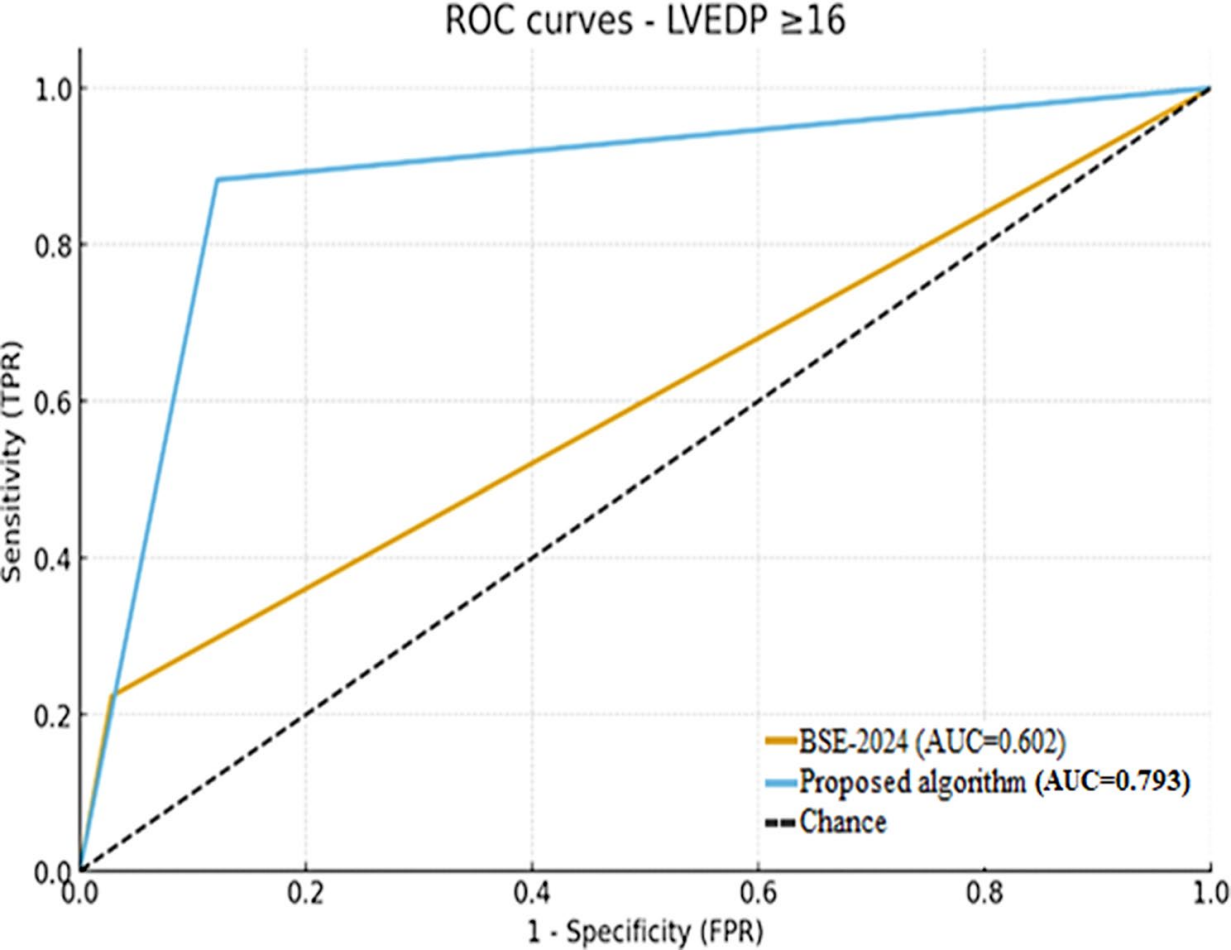
Area under the curve (AUC) comparison between BSE-2024 and proposed algorithm for LVEDP≥16mmHg (derivation cohort). *: proposed algorithm data are derived after excluding approximately 13.7% of (indeterminate cases), whereas the BSE-2024 algorithm retained nearly all patients (0.7% indeterminate cases). therefore, direct comparisons of performance metrics should be interpreted with caution

The complementary algorithm shifted more studies from normal to elevated with modest 13.7% indeterminate (Table 7). It improved diagnostic performance compared with BSE-2024 alone (Table 8). For elevated LVEDP, the AUC increased from 0.602 to 0.793, with sensitivity increasing from 27.8% to 78.8% and overall accuracy from 58.5% to 79.3% (Fig. 5).

**Table 7:** Distribution of pressure categories comparison between BSE-2024 and proposed algorithm (derivation cohort)

Recommendation	Normal	Indeterminate	Elevated
BSE-2024	327 (81.5%)	3 (0.7%)	71 (17.7%)
Proposed Algorithm	243 (60.6%)	55 (13.7%)	103 (25.7%)
P value	< 0.0001	< 0.0001	< 0.0001

BSE = British Society of Echocardiography

**Table 8:** Diagnostic performance comparison between BSE-2024 and proposed algorithm (derivation cohort) *

LV pressure wave	Model	Sens.	Spec.	PPV	NPV	Accuracy	AUC (95%CI)	AUCs *P* value
LVEDP ≥ 16 mmHg	BSE2024	27.8%	93.5%	83.1%	53.2%	58.5%	0.602 (0.545–0.659)	< 0.0001
	Proposed	78.8%	79.8%	81.3%	77.2%	79.3%	0.793 (0.747–0.839)	
LV pre-A > 15 mmHg	BSE2024	38.2%	92.1%	70.4%	75.2%	74.4%	0.652 (0.588–0.716)	0.024
	Proposed	85.5%	67.3%	58.3%	89.7%	73.7%	0.764 (0.711–0.817)	
LVFP either positive	BSE2024	29.5%	96.1%	90.1%	53.2%	59.8%	0.622 (0.569–0.675)	< 0.0001
	Proposed	80.3%	83.2%	84.9%	78.3%	81.6%	0.818 (0.776–0.86)	

BSE: British Society of Echocardiography, Sens: sensitivity, Spec: specificity, PPV: positive predictive value, NPV: negative predictive value, AUC: area under the curve and LVEDP: left ventricular end-diastolic pressure, LVFP: left ventricular filling pressure. *: proposed algorithm data are derived after excluding approximately 13.7% of (indeterminate cases), whereas the BSE-2024 algorithm retained nearly all patients (0.7% indeterminate cases). therefore, direct comparisons of performance metrics should be interpreted with caution

**Fig. 6:**
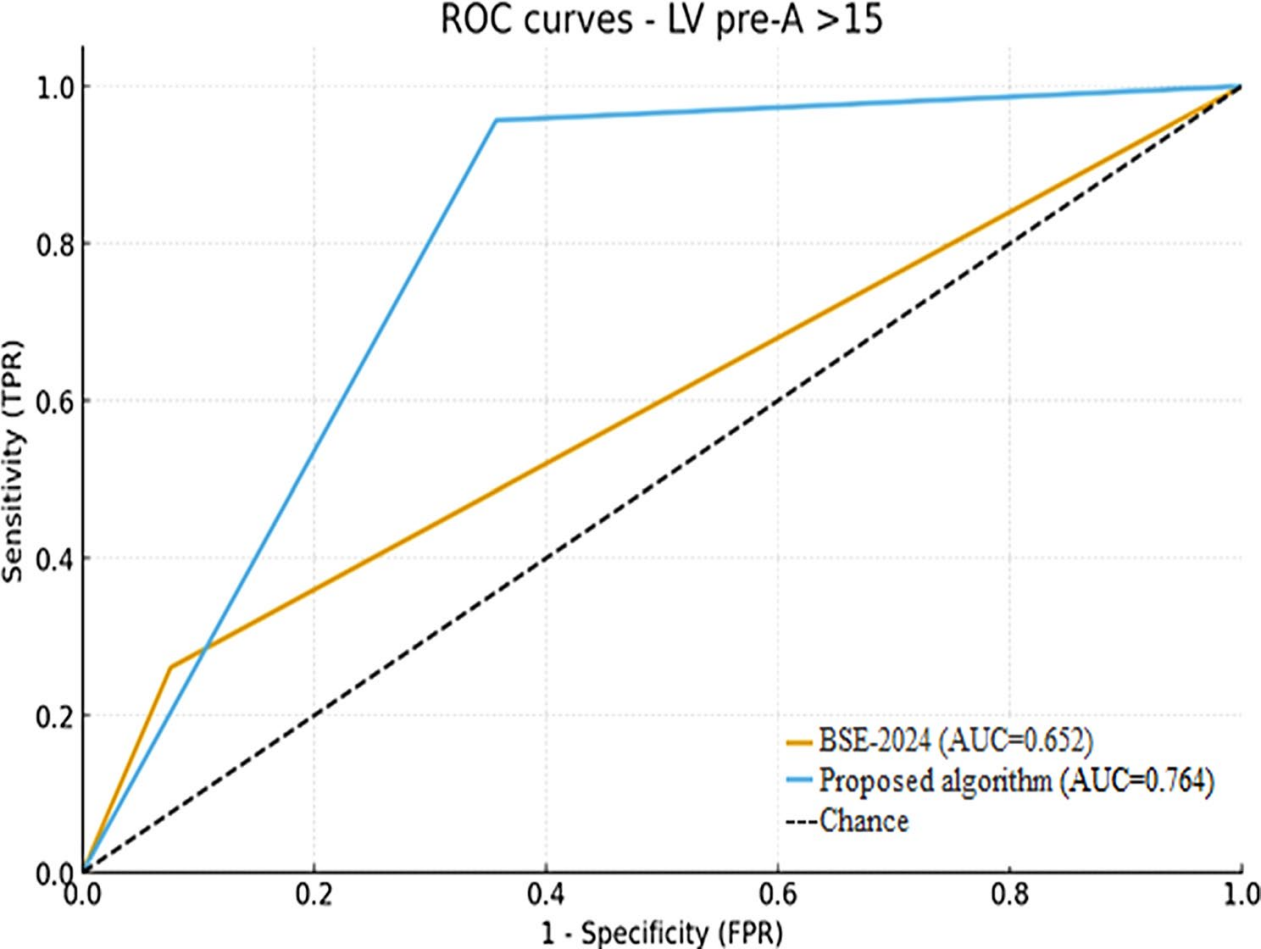
Area under the curve (AUC) comparison between BSE-2024 and proposed algorithm for LV pre-A>15mmHg (derivation cohort). *: proposed algorithm data are derived after excluding approximately 13.7% of (indeterminate cases), whereas the BSE-2024 algorithm retained nearly all patients (0.7% indeterminate cases). therefore, direct comparisons of performance metrics should be interpreted with caution

Consistent improvements were observed for LV pre-A pressure (AUC 0.652 to 0.764; sensitivity 38.2% to 85.5%; Fig. 6) and the exploratory LVFP endpoint (Supplementary Fig. 8). Reclassification and calibration analyses also favored the complementary algorithm (Supplementary Tables 5–7).

**Fig. 7:**
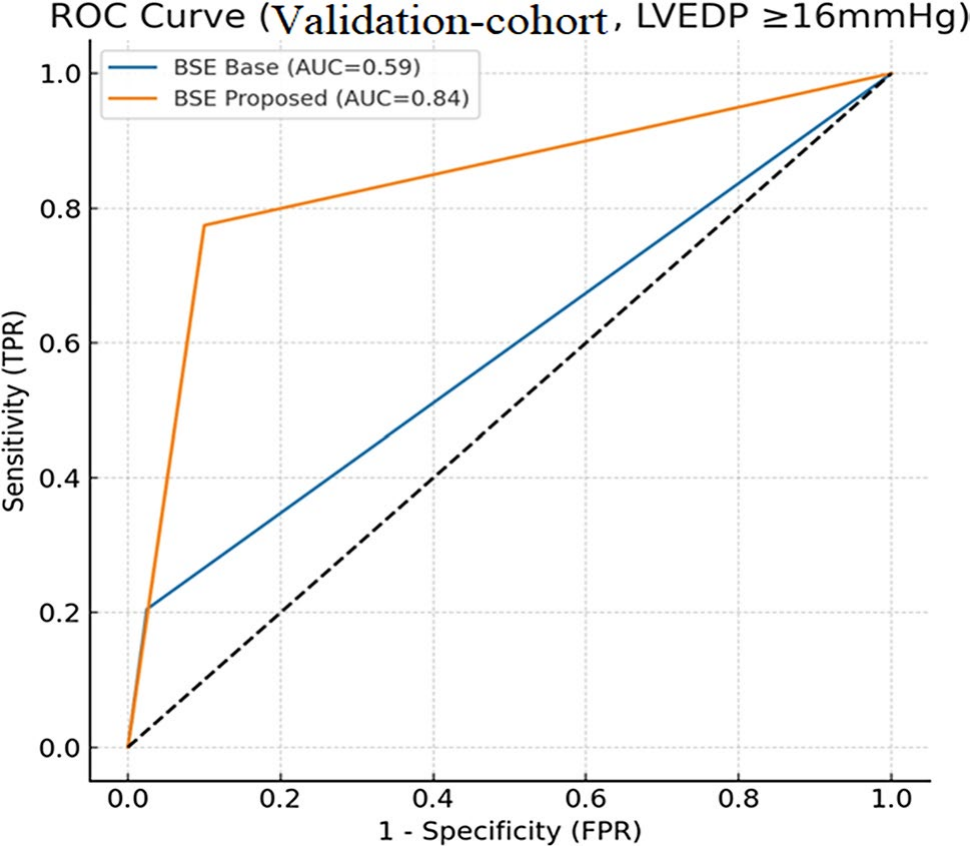
Validation-cohort area under the curve (AUC) comparison between BSE-2024 and proposed algorithm for LVEDP≥16mmHg. *: proposed algorithm data are derived after excluding approximately 9.2% of (indeterminate cases), whereas the BSE-2024 algorithm retained nearly all patients (0% indeterminate cases). Therefore, direct comparisons of performance metrics should be interpreted with caution

**Fig. 8:**
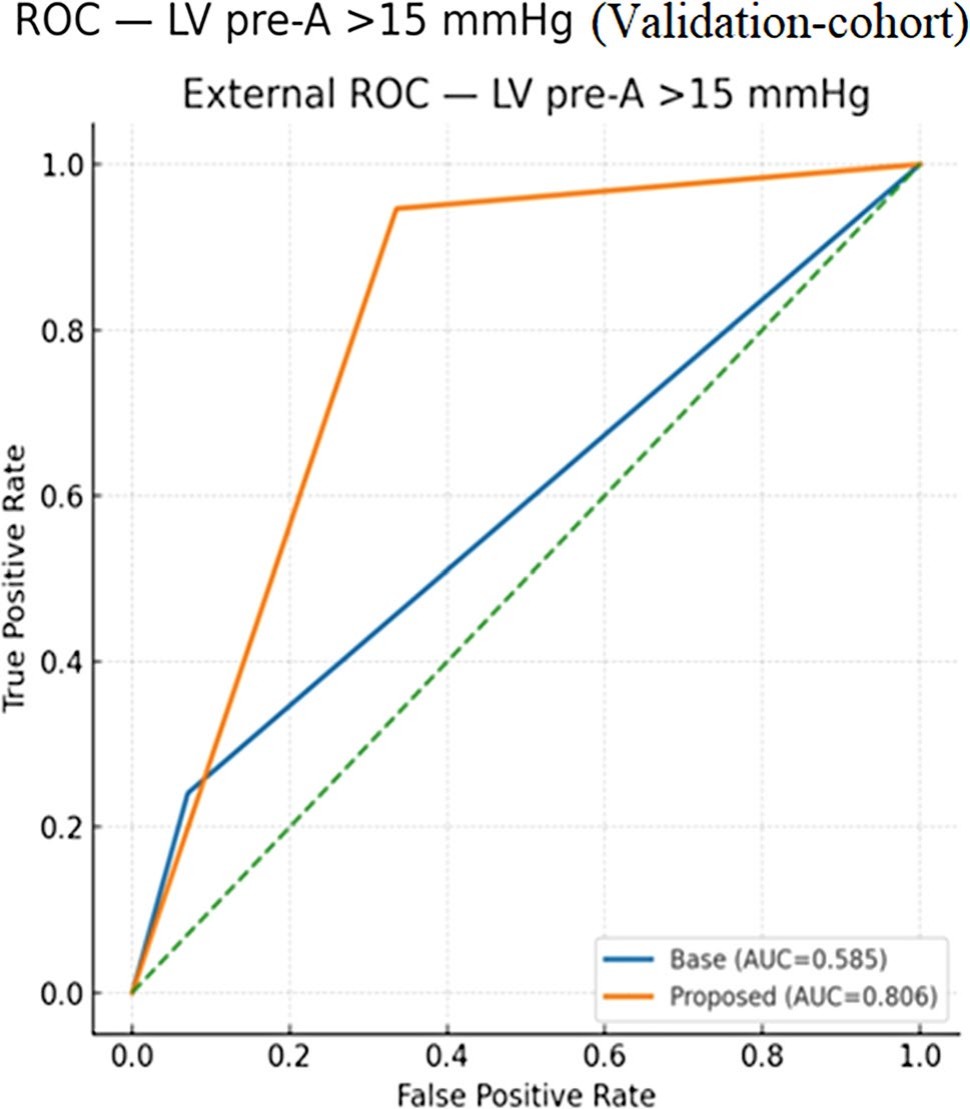
Validation-cohort area under the curve (AUC) comparison between BSE-2024 and proposed algorithm for LV pre-A15 > mmHg. *: proposed algorithm data are derived after excluding approximately 9.2% of (indeterminate cases), whereas the BSE-2024 algorithm retained nearly all patients (0% indeterminate cases). Therefore, direct comparisons of performance metrics should be interpreted with caution

The independent validation cohort confirmed the robustness and reproducibility of the proposed approach. The proposed algorithm consistently improved AUC (Figs. 7 and 8), sensitivity and overall diagnostic accuracy across all invasive reference standards (Tables 9 and 10) compared with BSE-2024 alone (Supplementary Fig. 9).

**Table 9:** LAP assessment comparison between BSE-2024 and proposed algorithm (validation-cohort)

Guideline	Indeterminate	Normal	Elevated
BSE-2024	0 (0.0%)	272 (86.3%)	43 (13.7%)
Proposed algorithm	29 (9.2%)	115 (36.5%)	171 (54.28%)
P-value	< 0.0001	< 0.0001	< 0.0001

BSE = British Society of Echocardiography

**Table 10:** Diagnostic performance comparison of validation-cohort between BSE-2024 and proposed algorithm*

LV pressure wave	Model	Sens.	Spec.	PPV	NPV	Accuracy	AUC	*P* value
LVEDP ≥ 16 mmHg	BSE2024	20.5%	97.5%	93.0%	43.0%	49.8%	0.59 (0.532–0.648)	< 0.0001
	Proposed	88.3%	87.9%	92.4%	81.7%	88.1%	0.84 (0.786–0.894)	
LV pre-A > 15 mmHg	BSE2024	25.6%	93.4%	69.8%	68.0%	68.3%	0.585 (0.522–0.648)	< 0.0001
	Proposed	95.7%	64.3%	64.3%	95.7%	76.9%	0.806 (0.745–0.867)	
LVFP either positive	BSE2024	20.4%	98.2%	95.3%	41.2%	48.6%	0.593 (0.544–0.642)	< 0.0001
	Proposed	87.5%	90.3%	94.2%	80%	88.5%	0.881 (0.812–0.908)	

BSE: British Society of Echocardiography, Sens: sensitivity, Spec: specificity, PPV: positive predictive value, NPV: negative predictive value, AUC: area under the curve and LVEDP: left ventricular end-diastolic pressure, LVFP: left ventricular filling pressure. *: proposed algorithm data are derived after excluding approximately 9.2% of (indeterminate cases), whereas the BSE-2024 algorithm retained nearly all patients (0% indeterminate cases). Therefore, direct comparisons of performance metrics should be interpreted with caution

## Discussion

This multicenter prospective study provides one of the first invasive comparative validations of the BSE-2024 and BSE-2013 algorithms in a derivation and an independent, temporally separated validation cohort.

BSE-2024 substantially improved classification feasibility, incorporating more than half of patients excluded by BSE-2013 as indeterminate cases. Despite this redistribution, updated guidelines maintained comparable but only modest diagnostic discrimination against invasive measurements. Comparisons should be interpreted cautiously because BSE-2013 excluded a large proportion of indeterminate cases, unlike BSE-2024, from diagnostic performance analyses. The proposed complementary algorithm improved sensitivity and overall discrimination, while maintaining acceptable specificity and relatively low rate of indeterminate classifications.

### Baseline findings

Baseline analyses demonstrated significant associations between invasive LVFP and both conventional and novel echocardiographic indices. Reduced LVEF, less negative GLS, lower LASr, larger LAVi and higher Doppler-derived indices were consistently associated with elevated LVFP, supporting the physiological validity of these parameters (Supplementary Fig. 10).

### Echocardiographic guidelines for diastolic function assessment

BSE-2013 classified more than half as indeterminate (60.6%), mainly due to discrepancy regarding E/A and EDT cutoff together in first step algorithm.

BSE-2024 shifted this classification to both impaired and normal group. This new classification showed a clearer relationship with invasive hemodynamic measurements for both LV pre-A and LVEDP, unlike BSE-2013 (Supplementary Table 8).

### Echocardiographic guidelines for LVFP assessment

BSE-2024 substantially reduced indeterminate classifications. This shift of reclassification predominantly affected normal group more than elevated group (Table 3).

While both guidelines showed important correlations with invasive means-LVFPs, BSE-2024 was statistically more significant (Supplementary Table 9).

### Invasive validation findings

The principal methodological finding is that direct diagnostic performance comparisons between BSE-2013 and BSE-2024 should be interpreted cautiously, because the 2013 algorithm excluded a large proportion of indeterminate cases before diagnostic performance was calculated. Therefore, these findings highlight the importance of interpreting comparisons between the two guideline algorithms in a broader clinical context rather than relying solely on direct diagnostic performance metrics.

After exclusion of the respective indeterminate cases, both BSE algorithms demonstrated comparable but modest discrimination (AUC) against invasive reference standards. Although BSE-2024 achieved higher specificity, this occurred at the expense of lower sensitivity. These findings suggest that BSE-2024 clinically improves confidence in ruling in elevated LVFP by increasing specificity; however, this occurs at the expense of reduced sensitivity.

Although BSE-2024 virtually eliminated indeterminate classifications, NRI and IDI analyses showed no significant improvement over BSE-2013 after excluding indeterminate cases. This apparent discrepancy reflects the fundamentally different classification strategies of the two recommendations rather than a true difference in diagnostic capability (Supplementary Table 10). Accordingly, the low agreement between algorithms suggests that they should not be considered directly interchangeable despite their comparable AUC values.

### Exploratory invasive method as reference standard

The exploratory composite invasive endpoint, based on either elevated LVEDP or LV pre-A pressure, was introduced to better reflect the multidimensional physiological concept of LVFP targeted by echocardiographic algorithms.

Elevated LVFP [21] is defined by at least one invasively-measured elevated wave (Supplementary Fig. 11). Thus, validation studies could cause a degree of bias when they depend only on one wave as an invasive reference standard. In contrast, echo-guidelines use a mixture of several echo-parameters that vary in relation with each invasively measured wave. However, echo-guidelines depend on mLAP as a non-invasive reference standard despite this mixture.

Gathering all together, this method was considered exploratory and requires further investigation (Supplementary Fig. 12).

### Feasibility of PV flow and TR parameters

In previous reports, PV feasibility varied widely (~ 65%-95%) [22–24]. Although current results aligned with experienced centers, this limitation may be challenging (as low as 65%) in real world practice [24]. However, recent recommendations emphasize their additive value when results are inconclusive but elevated LVFP is still suspected [8, 13].

### Complementary algorithm findings

Adding the complementary algorithm to BSE-2024 substantially improved sensitivity, overall discrimination (AUC), reclassification and diagnostic accuracy while maintaining acceptable specificity and relatively low rate of indeterminate classifications.

Importantly, these improvements were reproduced without modifying the algorithm in the temporally independent validation cohort, supporting the robustness of the proposed approach while still requiring further external validation before routine implementation.

### Literature review

The present findings are consistent with previous reports. In Luke et al. study [7] and an abstract of Barakat et al. [25], BSE-2013 was criticized for high indeterminate rates (near half of patients).

Tolvaj et al. [26] showed that BSE-2024 significantly reduced indeterminate (0.6%) and classified (97%) of patients as having normal LVFP, whereas only (2%) were classified having elevated LVFP. Although this was non-invasive assessment, the high-normal shift raises concerns regarding BSE-2024 sensitivity. Similar findings and concerns were observed in an abstract of Kolesnyk M et al. [27]. Furthermore, ~ 20% of hypertensive women had LASr in grey zone which may support additional parameters such as PV indices used in the complementary proposed algorithm [27]. In June 2025, Raja Shariff RE et al. [28] demonstrated statistically significant reduction in diastolic dysfunction group for BSE-2024 in comparison of ASE/EACVI 2016.

Although these studies were based on non-invasive assessment, the marked shift toward normal LVFP classification raises concerns regarding BSE-2024 sensitivity agreement with invasive LVFP. This is particularly noteworthy because this normal categorization was even greater than that reported using the EACVI/ASE 2016 recommendations which has been extensively studied and has been associated with suboptimal sensitivity.

Collectively, these studies suggest that BSE-2024 improves feasibility by reducing indeterminate classifications, but they also raise a consistent concern that this improvement may be accompanied by a shift toward normal LVFP classification and reduced sensitivity.

## Limitations

- First, this study is subject to spectrum bias because all the patients were referred to clinically indicated LHC. While the best method is to screen randomized patients, this would require additional ethical justification and broader approval to refer them for an invasive procedure that was not clinically indicated. These findings should be interpreted within a selected catheterization population and further studies with different populations are required to confirm generalizability to unselected outpatient cohorts.
- PV flow measurements were not feasible in all patients and some centers do not routinely perform these assessments, which may limit the applicability of the complementary algorithm.
- Echocardiography was performed shortly before, rather than during, LHC. Although simultaneous measurements would be ideal, no significant hemodynamic differences were observed, suggesting that this limitation had minimal impact on the results.
- Although the sample size was relatively large compared with previous validation studies, further external validation is required.
- Excluding indeterminate cases from the BSE-2013 algorithm may have introduced selection bias and could potentially overestimate its diagnostic performance. However, this reflects a known limitation of the 2013 recommendations and on the other hand this also highlights the ability of BSE-2024 to classify a substantially larger number of patients while maintaining comparable AUC and diagnostic performance to BSE-2013. To minimize this limitation, additional comparison methods were also applied such as invasive mean comparisons and classification distributions, this was performed to all patients, not only those eligible for ROC.
- Although the BSE-2024 guidelines include a dedicated algorithm for patients with atrial fibrillation, such patients were excluded from the study to maintain the homogeneity with the BSE-2013 algorithm, which does not provide specific guidance for AF. This limits the generalizability of our findings to patients with atrial fibrillation.
- Nevertheless, the prospective multicenter design, standardized invasive reference measurements, blinded assessment and independent validation cohort strengthen the overall validity of the findings.

## Conclusions

BSE-2024 substantially improves classification feasibility by nearly eliminating indeterminate cases but at the cost of reduced sensitivity. The complementary algorithm provides a more balanced diagnostic profile and may represent a promising adjunct to the current recommendations. These findings support cautious interpretation of direct comparisons between BSE-2013 and BSE-2024 and justify further external validation of the complementary algorithm before routine clinical implementation.

## Future directions

Future studies should focus on the invasive validation of the BSE-2024 recommendations and the proposed complementary algorithm in larger independent cohorts. Further research is also needed to define the role of emerging echocardiographic parameters in LVFP estimation.

## Supplementary Information

Below is the link to the electronic supplementary material.

Supplementary Material 1

## Data Availability

De-identified data and analytic code are available on reasonable request to the corresponding author, subject to institutional policies.

## Abbreviations

LV: Left ventricular
DF: Diastolic function
DD: Diastolic dysfunction
LVFP: Left ventricular filling pressure
BSE: British Society of Echocardiography
LVEDP: Left ventricular end-diastolic pressure
AUC: Area under the curve
HF: Heart failure
LA: Left atrial
ASE: American Society of Echocardiography
EACVI: European Association of Cardiovascular Imaging
EF: Ejection fraction
HFpEF: Heart failure with preserved ejection fraction
HFrEF: Heart failure with reduced ejection fraction
AF: Atrial fibrillation
GLS: Global longitudinal strain
LASr: Left atrial reservoir strain
LAVi: LA volume indexed to BSA
PCWP: Pulmonary capillary wedge pressure
LAP: Left atrial pressure
TTE: Transthoracic echocardiography
EDT: E wave of mitral flow deceleration time
TR: Tricuspid regurgitation
PASP: Pulmonary artery systolic pressure
PV: Pulmonary venous
ICC: Intra-class coefficients
PPV: Positive predictive value
NPV: Negative predictive value
DM: Diabetes mellitus
HTN: Hypertension
IHD: Ischemic heart disease
SD: Standard deviation
BMI: Body mass index
BSA: Body surface area
ROC: Receiver operating characteristic
IDI: Integrated discrimination improvement
NRI: Net reclassification improvement
