# Supplementary material for: Invasive validation of BSE-2024 vs. BSE-2013 using LVEDP and LV pre-A pressure in patients undergoing cardiac catheterization: a multicenter study with a complementary algorithm
Source: Echo Res Pract. 2026 Jul 27;13:27. doi: 10.1186/s44156-026-00132-4 (PMC13404105; doi:10.1186/s44156-026-00132-4)
Supplement: Supplementary file 1 — Supplementary Material 1 [file 44156_2026_132_MOESM1_ESM.docx]

***Supplemental Tables (1 to 10):***

| Table 1: Baseline characteristics by LVEDP ≥ 16 mmHg | | | |
| --- | --- | --- | --- |
| Descriptive Variables | | | |
| Variable | LVEDP <16 (n=187) | LVEDP ≥16 (n=214) | P-value |
| HTN | 104 (55.6%) | 111 (51.9%) | 0.5157 |
| DM | 70 (37.4%) | 150 (70.1%) | <0.0001 |
| IHD | 73 (39%) | 124 (57.9%) | 0.0002 |
| Female | 104 (55.6%) | 80 (37.4%) | 0.0004 |
| Continuous Variables | | | |
| Variable | LVEDP<16 Mean±SD | LVEDP≥16 Mean±SD | P-value |
| Age (years) | 54.1 ± 10.33 | 56.6 ± 7.37 | 0.0063 |
| EF (%) | 0.58 ± 0.09 | 0.55 ± 0.09 | 0.0016 |
| LAVI (mL/m²) | 20.18 ± 6.95 | 23.86 ± 9.29 | <0.0001 |
| Average E/e′ (unitless) | 7.76 ± 3.60 | 8.6 ± 3.86 | 0.0242 |
| LA Strain (%) | 30.87 ± 10.37 | 25 ± 9.69 | <0.0001 |
| GLS (%) | -18.22 ± 1.54 | -15.65 ± 1.41 | <0.0001 |
| E/A (ratio) | 1.01 ± 0.33 | 1.13 ± 0.41 | 0.0042 |
| DT (ms) | 207.94 ± 50.43 | 190.87 ± 48.4 | 0.0001 |
| TR Velocity (m/s) | 1.53 ± 0.7 | 1.9 ± 0.94 | 0.0002 |
| PASP (mmHg) | 23.17 ± 10 | 35.6 ± 16.56 | 0.0002 |
| S/D Ratio | 1.48 ± 0.39 | 1.28 ± 0.38 | <0.0001 |
| Ar-A duration (ms) | 13.05 ± 22.26 | 25.62 ± 16.05 | <0.0001 |
| BMI (kg/m²) | 27.93 ± 4.98 | 28.48 ± 4.77 | 0.2390 |
| BSA (m²) | 1.92 ± 0.19 | 1.93 ± 0.19 | 0.6700 |

LVEDP: Left Ventricular End-Diastolic Pressure, SD: standard deviation, EF: Ejection Fraction, LAVI: Left Atrial Volume Index, average E/e′: Mitral inflow E to Tissue Doppler e′ velocity (averaged), LA strain: Left Atrial Reservoir Strain (%), GLS: Global Longitudinal Strain, E/A: Ratio of early to late mitral inflow velocity, DT: Deceleration Time of E mitral flow, TR: Tricuspid Regurgitation velocity, PASP: Pulmonary Artery Systolic Pressure, S/D: Pulmonary Vein Systolic/Diastolic Ratio, Ar-A: Difference in duration between pulmonary vein Ar and mitral A wave, BMI: Body Mass Index and BSA: Body Surface Area, HTN: Hypertension, DM: diabetes mellitus and IHD: ischemic heart disease.

| Table 2: Hemodynamic Comparison (Echocardiography vs Catheterization) | | | |
| --- | --- | --- | --- |
| Variable | Echo Mean ± SD | Cath Mean ± SD | P-value |
| Heart Rate (bpm) | 72 ± 10 | 74 ± 11 | 0.42 |
| Systolic BP (mmHg) | 130 ± 15 | 132 ± 14 | 0.55 |
| Diastolic BP (mmHg) | 75 ± 10 | 76 ± 9 | 0.61 |
| Mean Arterial Pressure (mmHg) | 93 ± 11 | 95 ± 10 | 0.47 |

*Comparison of key hemodynamic parameters measured during echocardiography vs cardiac catheterization. Values are presented as Mean ± SD.*

| Table 3: Symptoms, drugs and other baseline characteristics | |
| --- | --- |
| Characteristic | (%) |
| Chronic Kidney Disease (eGFR <60) | (17.3%) |
| Beta-blockers | (58.5%) |
| ACEi/ARB | (51.7%) |
| Diuretics | (35.5%) |
| Statins | (54.9%) |
| Dyspnea (NYHA ≥ II) | (52.7%) |
| Orthopnea | (17.5%) |
| Fatigue | (41.8%) |

NYHA: New York Heart Association.

| **Table 4: LAP and diastolic function assessment agreement (BSE: 2013 vs 2024)*** | | | |
| --- | --- | --- | --- |
| Method | **Metric** | Estimate | P value |
| Diastolic function assessment | **Cohen’s kappa** | 0.133 | 0.0026 |
|  | **Agreement** | 43.2% | - |
| LAP assessment | **Cohen’s kappa** | 0.284 | 0.0289 |
|  | **Agreement** | 72.9% | - |

*LAP: left atrial pressure, BSE: British Society of Echocardiography. *: BSE-2013 data are derived after excluding approximately 60% of (indeterminate cases), whereas the BSE-2024 algorithm retained nearly all patients. therefore, direct comparisons of performance metrics should be interpreted with caution.*

| Table 5: Derivation cohort cross-tabulation: Proposed vs BSE-2024 (LAP assessment) | | | |
| --- | --- | --- | --- |
| Proposed ↓ / BSE-2024 → | Normal | Elevated | Indeterminate |
| Normal | 184 | 0 | 0 |
| Elevated | 121 | 71 | 0 |
| Indeterminate | 22 | 0 | 3 |

*LAP: left atrial pressure, BSE: British Society of Echocardiography.*

| Table 6: NRI and IDI BSE-2024 vs proposed algorithm for different invasive pressure wave (derivation cohort) | | | | |
| --- | --- | --- | --- | --- |
| LV pressure wave | Metric | Estimate | 95% CI | p-value |
| LVEDP ≥16 mmHg | Event NRI | +0.49 | 0.42 to 0.55 | p<0.001 |
|  | Non-event NRI | −0.135 | −0.19 to −0.09 | p<0.001 |
|  | Total NRI | +0.355 | 0.27 to 0.44 | p<0.001 |
|  | IDI (Δ discrimination slope) | +0.355 | 0.27 to 0.44, | p<0.001 |
| LV pre-A >15 mmHg | Event NRI | +0.473 | 0.39 to 0.56 | p<0.001 |
|  | Non-event NRI | −0.241 | −0.30 to −0.19 | p<0.001 |
|  | Total NRI | +0.232 | 0.14 to0.33 | p<0.001 |
|  | IDI (Δ discrimination slope) | +0.232 | 0.14 to 0.33 | p<0.001 |
| LVFP either positive | Event NRI | +0.488 | 0.42 to 0.55 | p<0.001 |
|  | Non-event NRI | −0.127 | −0.18 to −0.08 | p<0.001 |
|  | Total NRI | +0.361 | 0.27 to 0.44 | p<0.001 |
|  | IDI (Δ discrimination slope) | +0.361 | 0.27 to 0.44, | p<0.001 |

*NRI Net Reclassification Index and IDI: Integrated Discrimination Improvement.*

| Table 7: Derivation calibration between BSE-2024 and proposed algorithm * | | | | |
| --- | --- | --- | --- | --- |
| Endpoint | **Model** | **Observed % Normal** | **Observed % Elevated** | **Brier score** |
| LVEDP ≥16mmHg | BSE2024 | 46.8% | 83.1% | 0.415 |
|  | Proposed | 22.8% | 81.2% | 0.207 |
| LV pre-A >15mmHg | BSE2024 | 24.8% | 70.4% | 0.256 |
|  | Proposed | 10.3% | 58.3% | 0.263 |
| LVFP positive | BSE2024 | 46.8% | 90.1% | 0.402 |
|  | Proposed | 21.7% | 84.9% | 0.184 |

*BSE: British Society of Echocardiography, LVEDP: left ventricular end-diastolic pressure, LVFP: left ventricular filling pressure. *: proposed algorithm data are derived after excluding approximately 13.7% of (indeterminate cases), whereas the BSE-2024 algorithm retained nearly all patients (0.7% indeterminate cases). therefore, direct comparisons of performance metrics should be interpreted with caution.*

| Table 8: Means comparison of invasive pressures by diastolic function classification (BSE-2013 vs BSE-2024) | | | | |
| --- | --- | --- | --- | --- |
| Guidelines | Group | Normal | Impaired diastolic function | P-value (comparison) |
| BSE-2013 | Mean LVEDP ±SD (mmHg) | 17.1 ± 6.8 | 16.55 ± 5.9 | 0.6458 |
|  | Mean LV pre-A ± SD (mmHg) | 10.8 ± 5.2 | 12.45 ± 5.27 | 0.1021 |
| BSE-2024 | Mean LVEDP ±SD (mmHg) | 16.2 ± 5.9 | 19.8 ± 6.5 | <0.00001 |
|  | Mean LV pre-A ± SD (mmHg) | 11.0 ± 4.6 | 14.2 ± 5.3 | <0.00001 |

*BSE = British Society of Echocardiography, LVEDP: left ventricular end diastolic pressure.*

| Table 9: Comparison of invasive pressures by LAP classification (BSE-2013 vs BSE-2024)* | | | | |
| --- | --- | --- | --- | --- |
| Guidelines | Group | Normal LAP | Elevated LAP | P-value (comparison)* |
| BSE-2013 | Mean LVEDP ±SD (mmHg) | 16.85 ± 6.34 | 20.5 ± 6.25 | 0.0009 |
|  | Mean LV pre-A ± SD (mmHg) | 11.6 ± 5.3 | 15.05 ± 5.57 | 0.0003 |
| BSE-2024 | Mean LVEDP ±SD (mmHg) | 17.4 ± 6.5 | 21.2 ± 5.5 | <0.00001 |
|  | Mean LV pre-A ± SD (mmHg) | 11.6 ± 4.8 | 17 ± 4.6 | <0.00001 |

*BSE = British Society of Echocardiography, LVEDP: left ventricular end diastolic pressure. *: BSE-2013 data are derived after excluding approximately 60% of (indeterminate cases), whereas the BSE-2024 algorithm retained nearly all patients. therefore, direct comparisons of performance metrics should be interpreted with caution.*

| **Table 10: Calibration results (Observed invasive vs Predicted LAP codes)*** | | | | | |
| --- | --- | --- | --- | --- | --- |
| LV pressure wave | Guidelines | Predicted Normal | Predicted Elevated* | Observed Positive | Brier score |
| Observed LVEDP≥16 mmHg | BSE-2013 | 67.7% | 32.3% | 54.4% | 0.386 |
|  | BSE-2024 | 82.2% | 17.8% | 63.6% | 0.415 |
| Observed LV pre-A>15 mmHg | BSE-2013 | 67.7% | 32.3% | 38.6% | 0.316 |
|  | BSE-2024 | 82.2% | 17.8% | 32.9% | 0.256 |
| Observed LVFP either positive | BSE-2013 | 67.7% | 32.3% | 56.3% | 0.392 |
|  | BSE-2024 | 82.2% | 17.8% | 54.5% | 0.402 |

*BSE: British Society of Echocardiography, LVEDP: left ventricular end diastolic pressure, LVFP: left ventricular filing pressure. BSE-2013 data are derived after excluding approximately 60% of (indeterminate cases), whereas the BSE-2024 algorithm retained nearly all patients. therefore, direct comparisons of performance metrics should be interpreted with caution.*

***Supplemental figures (1 to 12):***

*
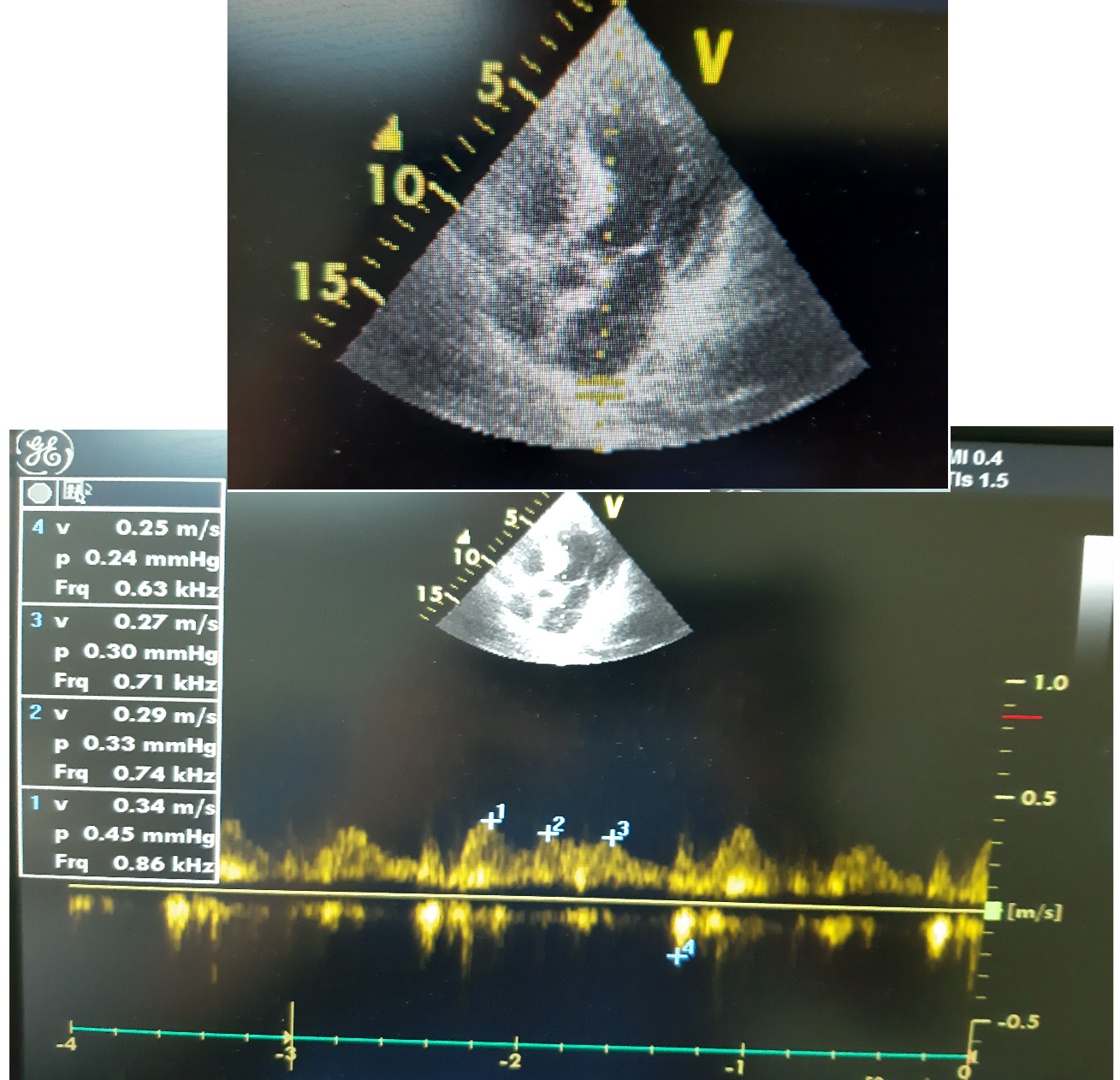
*

*Figure 1: pulmonary veins measurements: first 2D mode and second: Doppler mode.*

*
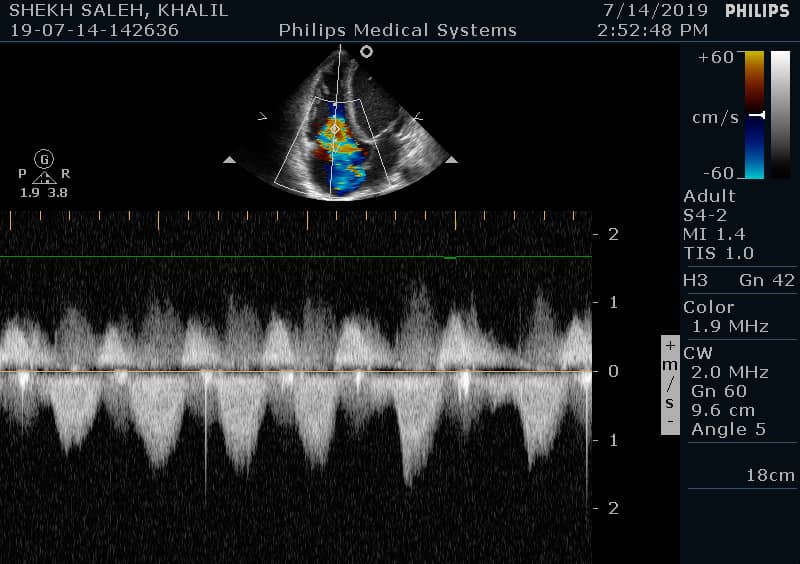
*

*Figure 2: example of echo measurements.*

*
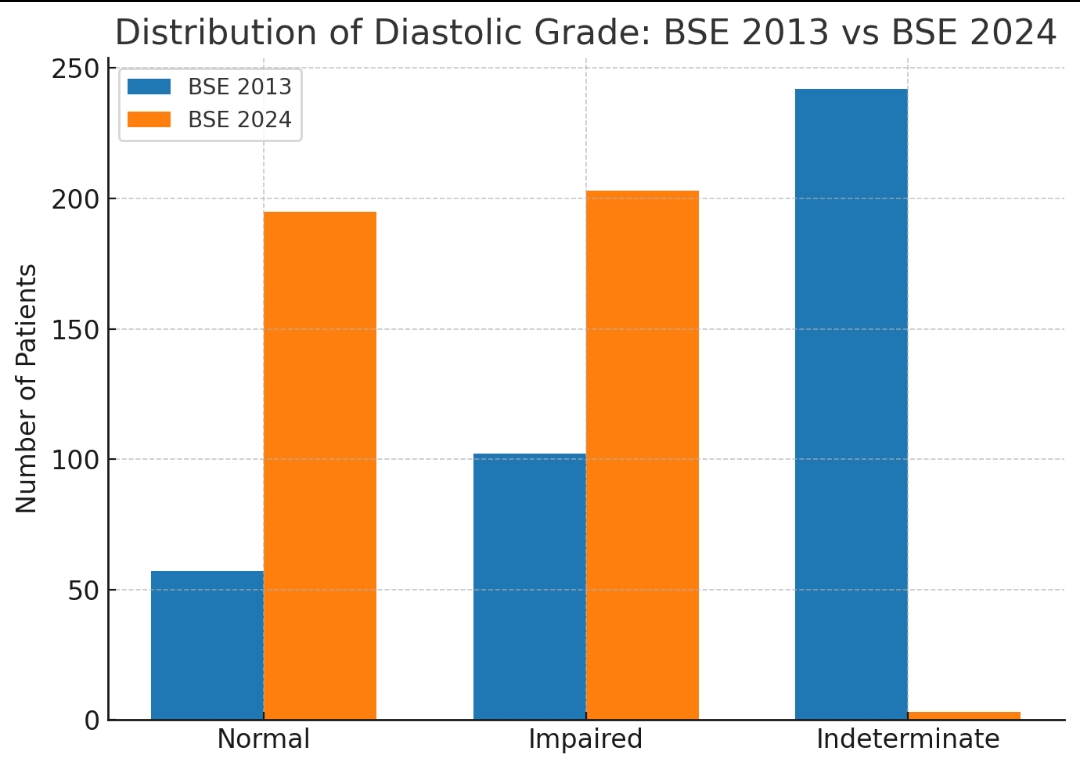
*

*Figure 3: Diastolic function grades distribution comparison between BSE-2013 and BSE-2024.*

*
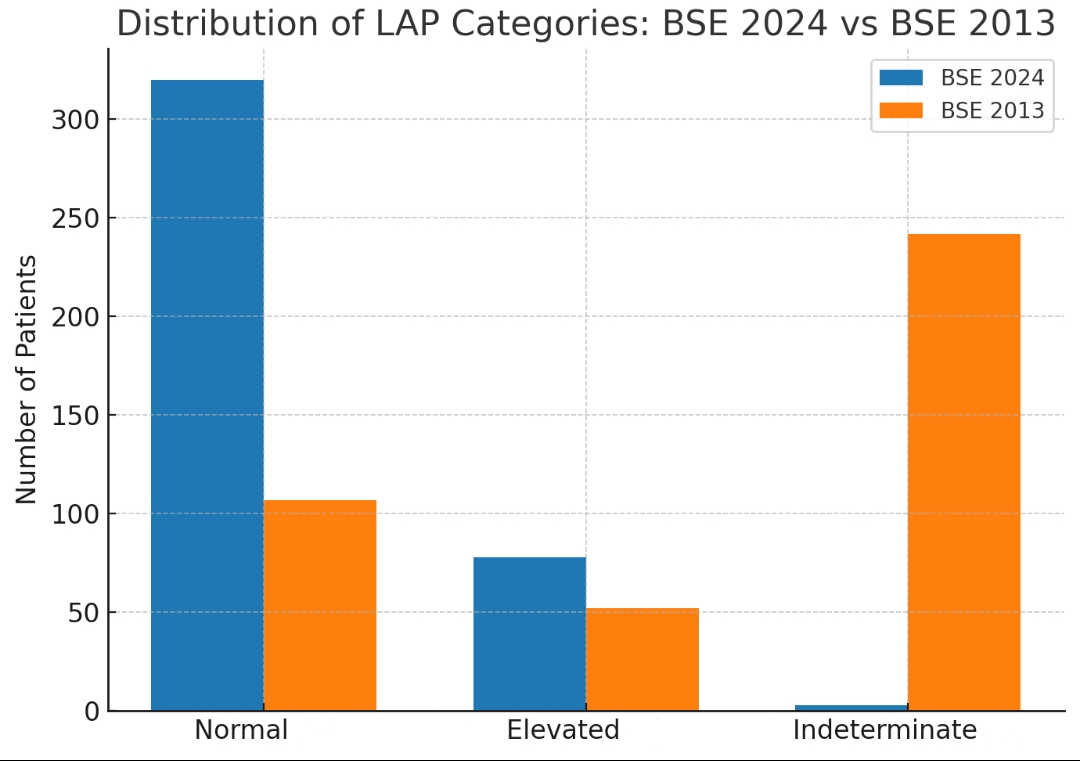
*

*Figure 4: Left atrial pressure assessment distribution comparison between BSE-2013 and BSE-2024.*

*
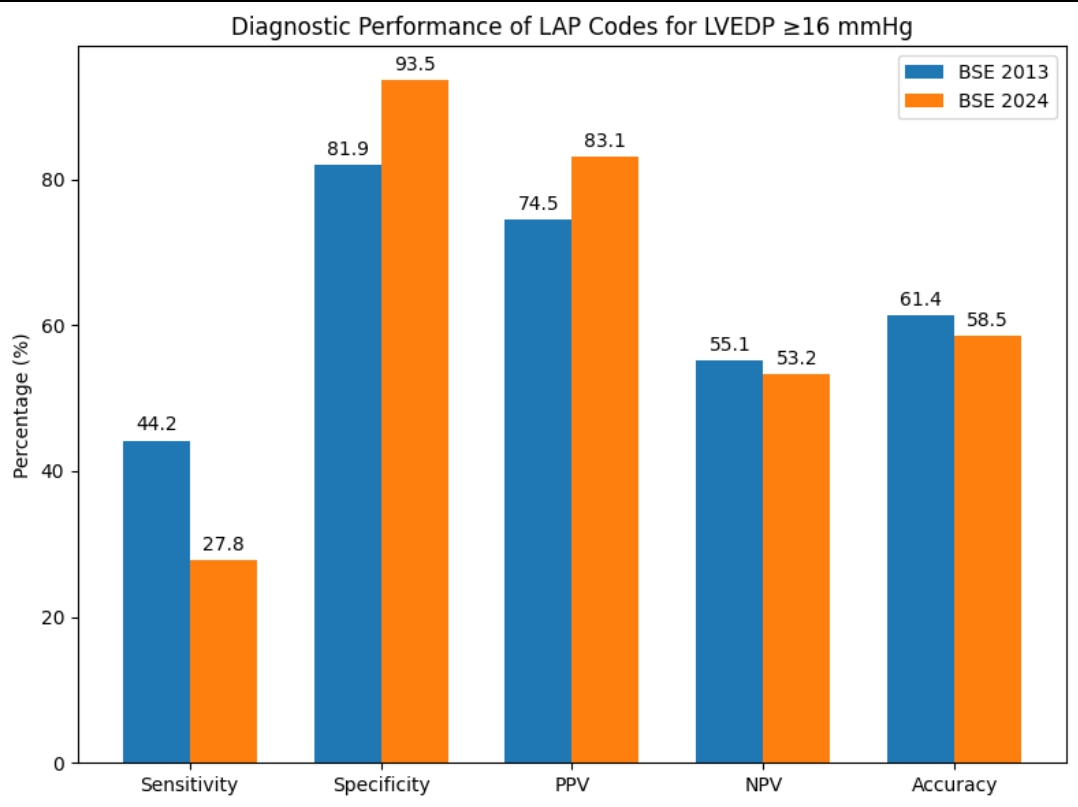
*

*Figure 5: Diagnostic performance comparison between BSE-2013 and BSE-2024 for LVEDP≥16mmHg. *: BSE-2013 data are derived after excluding approximately 60% of (indeterminate cases), whereas the BSE-2024 algorithm retained nearly all patients. therefore, direct comparisons of performance metrics should be interpreted with caution.*

*
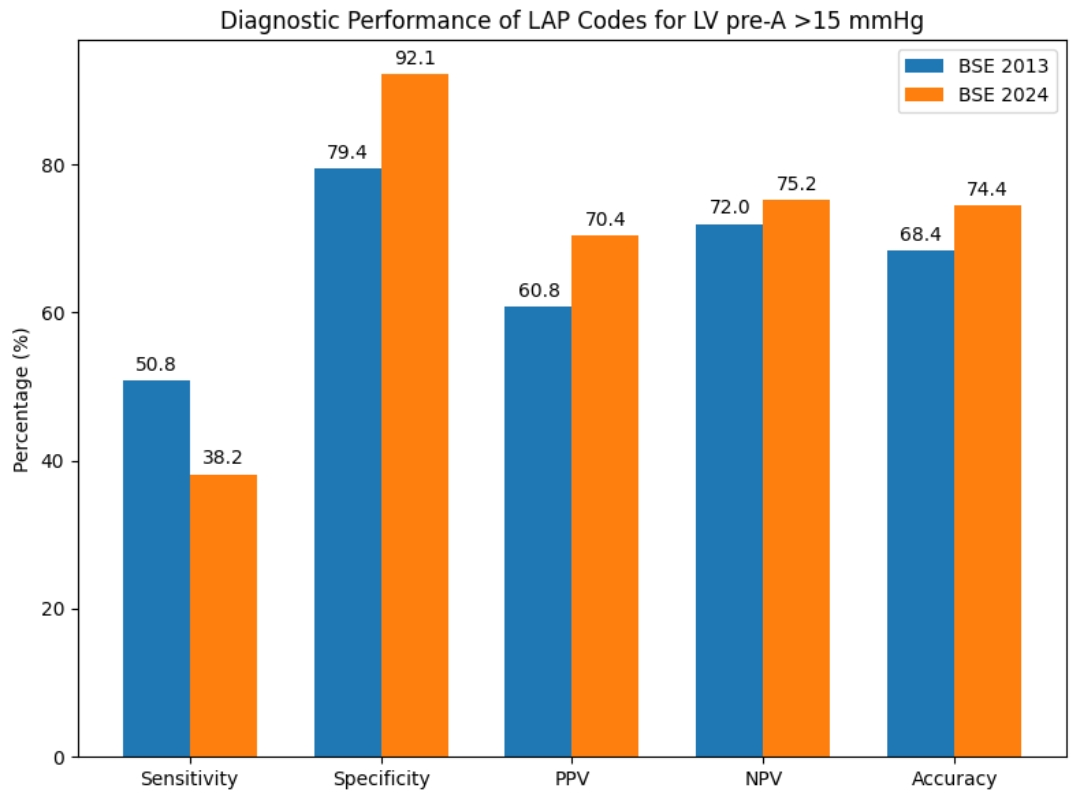
*

*Figure 6: Diagnostic performance comparison between BSE-2013 and BSE-2024 for LV pre-A>15mmHg. *: BSE-2013 data are derived after excluding approximately 60% of (indeterminate cases), whereas the BSE-2024 algorithm retained nearly all patients. therefore, direct comparisons of performance metrics should be interpreted with caution.*

*
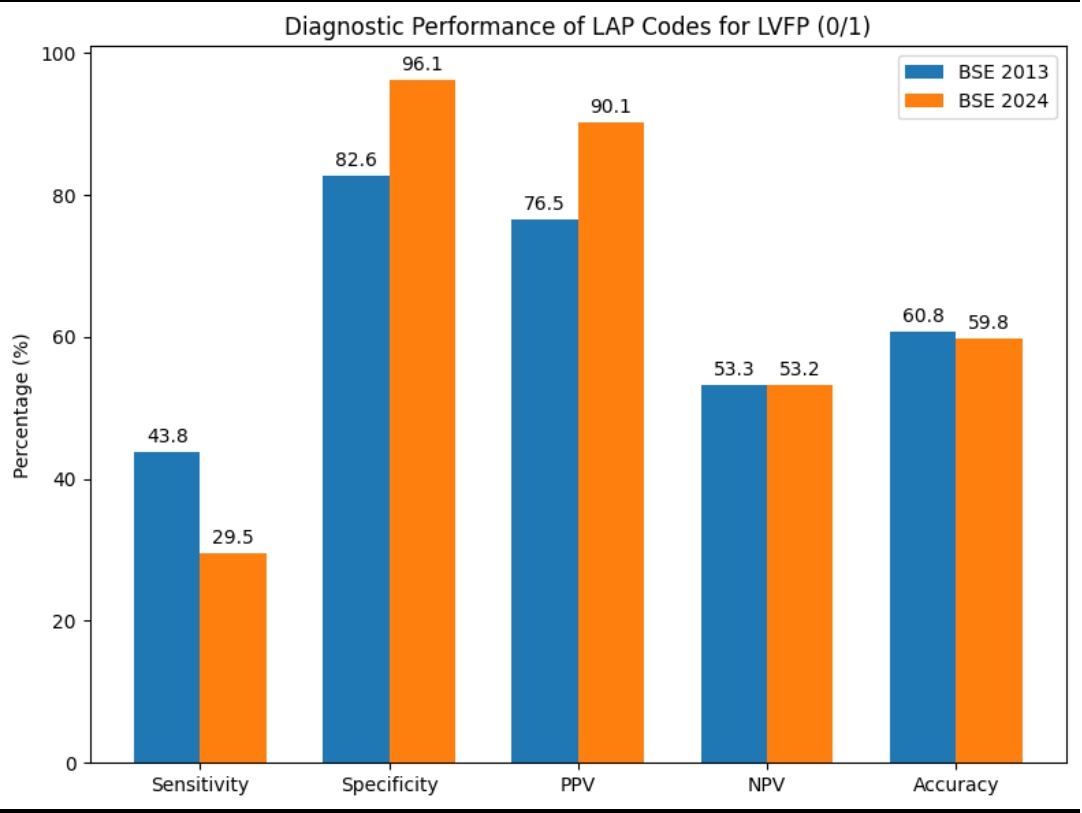
*

*Figure 7: Diagnostic performance comparison between BSE-2013 and BSE-2024 for LVFP either positive (LVEDP≥16mmHg or LV pre-A>15mmHg). *: BSE-2013 data are derived after excluding approximately 60% of (indeterminate cases), whereas the BSE-2024 algorithm retained nearly all patients. therefore, direct comparisons of performance metrics should be interpreted with caution.*

*
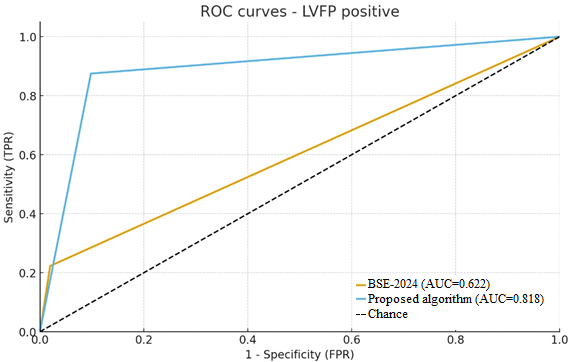
*

*Figure 8: Area under the curve (AUC) comparison between BSE-2024 and proposed algorithm for LVFP either positive (LVEDP≥16mmHg or pre-A>15mmHg) in derivation cohort. *: proposed algorithm data are derived after excluding approximately 13.7% of (indeterminate cases), whereas the BSE-2024 algorithm retained nearly all patients (0.7% indeterminate cases). therefore, direct comparisons of performance metrics should be interpreted with caution.*

*
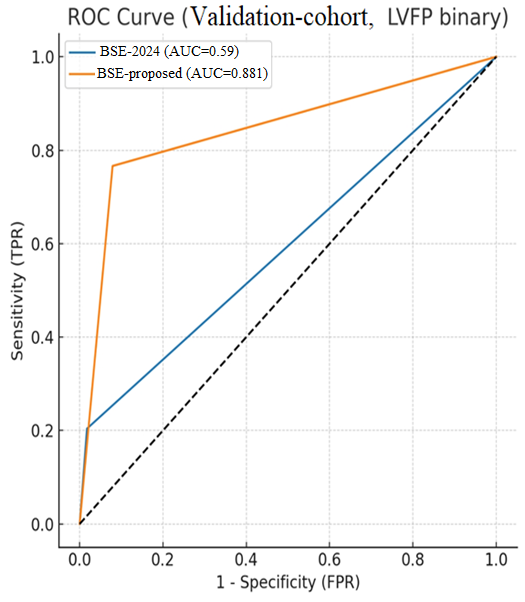
*

*Figure 9: Validation-cohort area under the curve (AUC) comparison between BSE-2024 and proposed algorithm for LVFP either positive (LVEDP≥16mmHg or pre-A>15mmHg). *: proposed algorithm data are derived after excluding approximately 9.2% of (indeterminate cases), whereas the BSE-2024 algorithm retained nearly all patients (0% indeterminate cases). Therefore, direct comparisons of performance metrics should be interpreted with caution.*

*
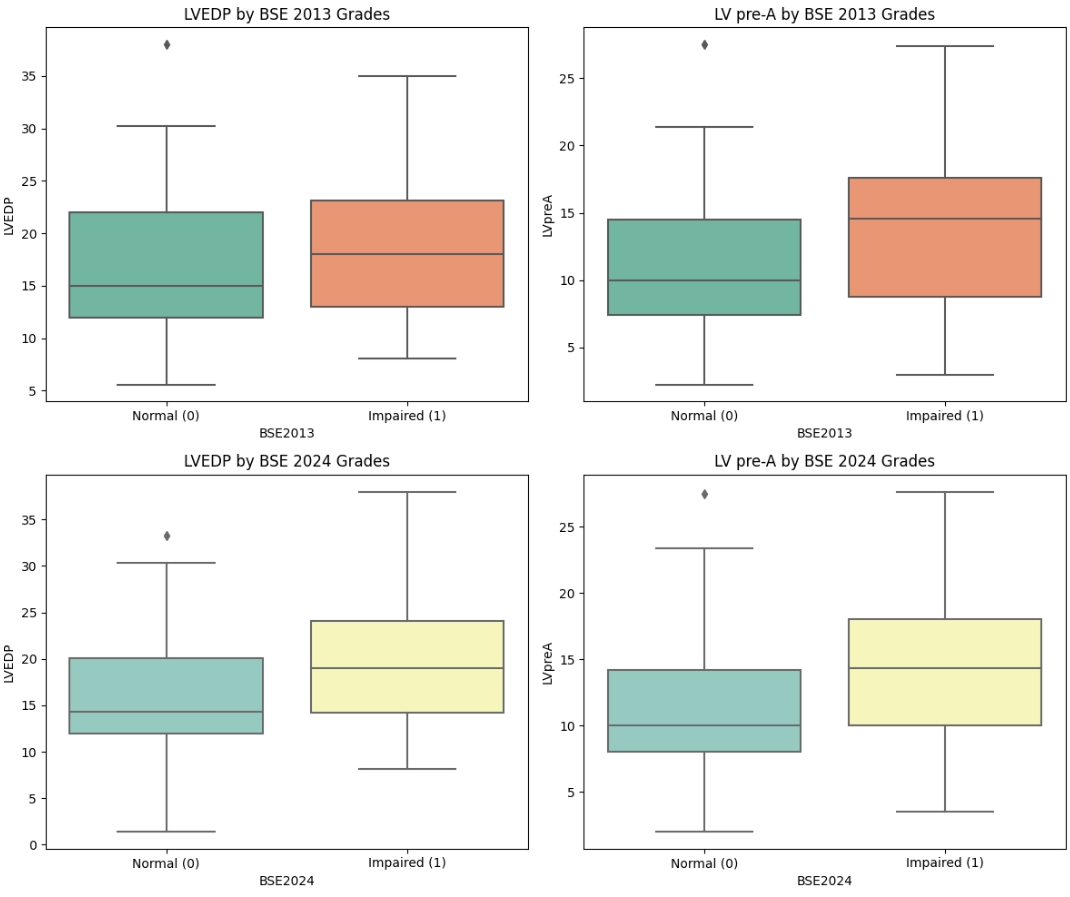
*

*Figure 10: Invasive LV filling pressure mean comparison between BSE-2024 and BSE-2013 for LVEDP≥16mmHg and LV pre-A>15mmHg.*

*
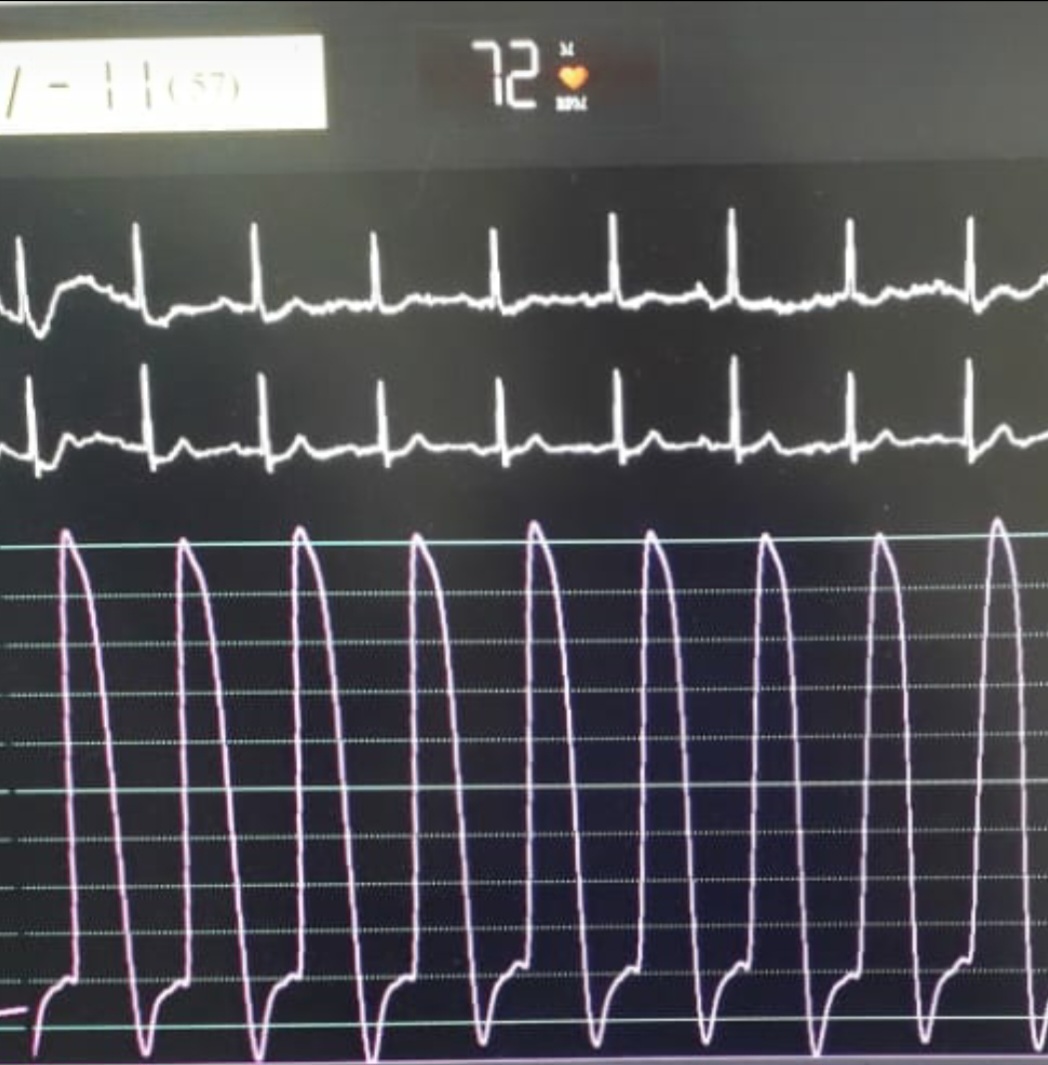
*

*Figure 11: example of invasive left ventricular filling pressure measurement.*

*
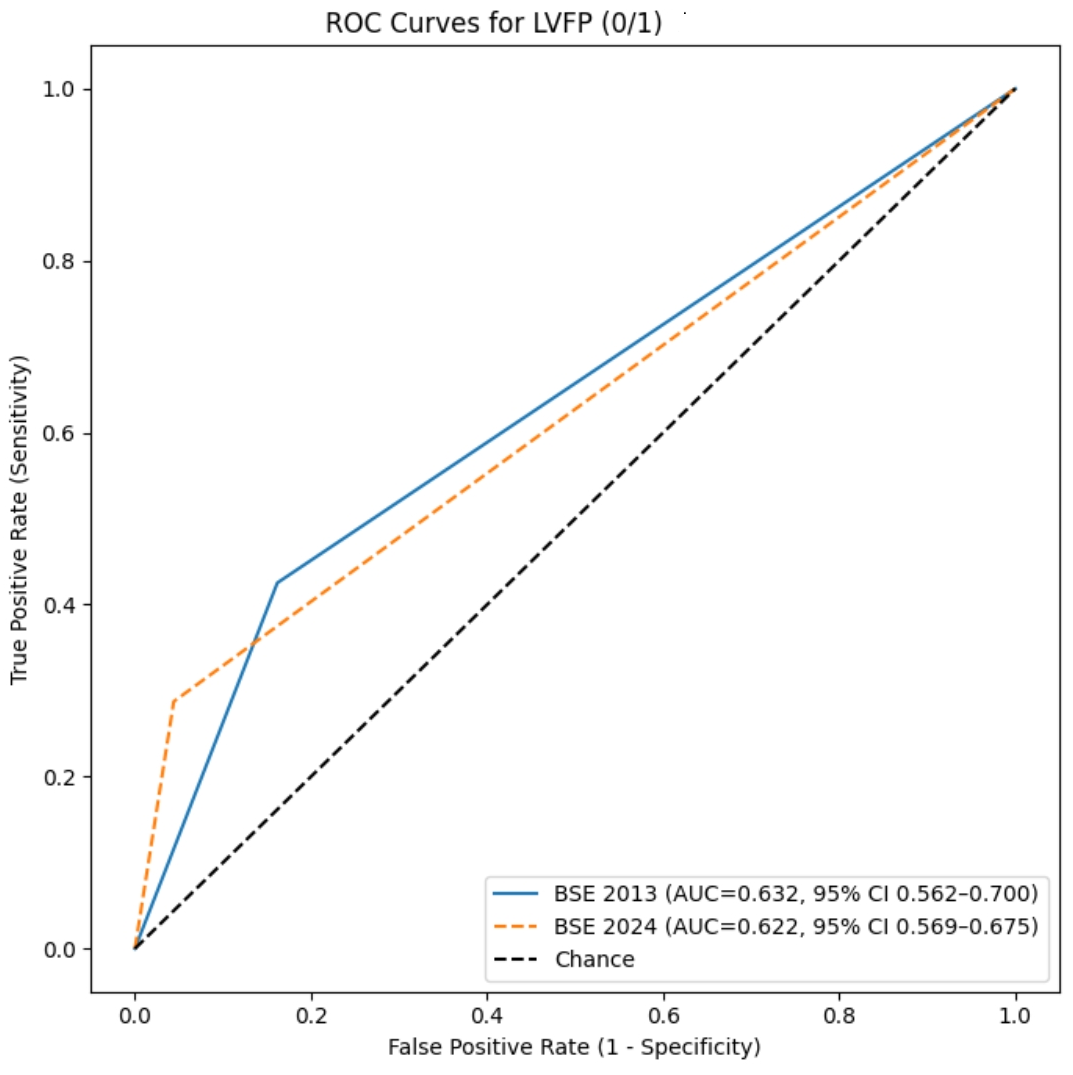
*

*Figure 12: Area under the curve (AUC) comparison between BSE-2013 and BSE-2024 for LVFP either positive (LVEDP≥16mmHg or pre-A>15mmHg). *: BSE-2013 data are derived after excluding approximately 60% of (indeterminate cases), whereas the BSE-2024 algorithm retained nearly all patients. therefore, direct comparisons of performance metrics should be interpreted with caution.*

*.*
